# Putative novel outer membrane antigens multi-epitope DNA vaccine candidates identified by Immunoinformatic approaches to control *Acinetobacter baumannii*

**DOI:** 10.1186/s12865-023-00585-w

**Published:** 2023-11-18

**Authors:** Niloofar Sadat Tabibpour, Abbas Doosti, Ali Sharifzadeh

**Affiliations:** 1 Department of Biology, Faculty of Basic Sciences, Shahrekord Branch, Islamic Azad University, Shahrekord, Iran; 2 Biotechnology Research Center, Shahrekord Branch, Islamic Azad University, Shahrekord, Iran; 3 Department of Microbiology, Faculty of Veterinary Medicine, Islamic Azad University, Shahrekord Branch, Shahrekord, Iran

**Keywords:** Multi-epitope polypeptide, Reverse vaccinology, Acinetobacter baumannii

## Abstract

Multi-epitope polypeptide vaccines, a fusion protein, often have a string-of-beads system composed of various specific peptide epitopes, potential adjuvants, and linkers. When choosing the sequence of various segments and linkers, many alternatives are available. These variables can influence the vaccine’s effectiveness through their effects on physicochemical properties and polypeptide tertiary structure.

The most conserved antigens were discovered using BLASTn. To forecast the proteins’ subcellular distribution, PSORTb 3.0.2 was used. Vaxign was used for the preliminary screening and antigenicity assessment. Protein solubility was also predicted using the ccSOL omics. Using PRED-TMBB, it was anticipated that the protein would localize across membranes. The IEDB and BepiPred-2.0 databases were used to predict the immunogenicity of B cell epitopes. A multi-epitope construct was developed and analyzed to evaluate. Twenty epitopes from *A. baumannii*’s outer membrane protein (omp) were included in the vaccination. TLR4 agonist explosibility was investigated. The physicochemical characteristics, secondary and tertiary structures, and B-cell epitopes of vaccine constructs were assessed. Additionally, docking and MD experiments were used to examine the relationship between TLR4 and its agonist.

Thirteen antigens were discovered, and eight of the 13 chosen proteins were predicted to be surface proteins. The 34 kDa outer membrane protein, Omp38, Omp W, CarO, putative porin, OmpA, were chosen as having the right antigenicity (≥0.5). FhuE and CdiA were eliminated from further study because of their low antigenicity. The vaccine design was developed by combining the most effective 10 B-cell and 10 MHC-I/MHCII combined coverage epitopes. The molecular formula of the vaccine was determined to be C1718H2615N507O630S17. The vaccine form has a molecular weight of 40,996.70 Da and 47 negatively charged residues (Asp + Glu), whereas 28 positively charged residues (Arg + Lys). The estimated half-life was 7.2 hours (mammalian reticulocytes, in vitro), > 20 hours (*yeast*, in vivo) and > 10 hours (*Escherichia coli*, in vivo) for the vaccine. The multi-epitope vaccine insertion is carried via the expression vector pcDNA3.1 (+).

The multi-epitope vaccine may stimulate humoral and cellular immune responses, according to our findings, and it may be a candidate for an *A. baumannii* vaccine.

**Supplementary Information:**

The online version contains supplementary material available at 10.1186/s12865-023-00585-w.

## Introduction

Genetic Modification has led to a significant advancement in pharmacological science by making it possible to produce a wide range of native and fusion proteins. Fusion peptides with various uses and activities can be produced by genetically combining two or more genes that produce distinct proteins. Drug-derived fusion peptides often include at least one domain with a primary therapeutic purpose, such as interacting with the relevant ligand [1]. Other fused components are also included for various purposes, such as enhancing molecular properties like stability and half-life or supporting roles, such as adjuvants in vaccines. The fused fragments will have the same bioavailability of drugs and distribution unless proteolytic enzymes break them down [2]. Fusion might also create innovative formations not seen in biology [1, 2].

Multi-epitope polypeptide vaccines, composed of multiple epitopic areas, garner significant interest compared to conventional vaccines due to their benefits, including excellent safety, higher stability, less inflammatory and autoimmunity reactions, and more efficient manufacture. Combination therapy is required to overcome the main drawback of multi-epitope polypeptide vaccines, which is their poor immunogenicity [3]. Because the adjuvant is delivered to the same antigen presentation cells (APC) and receptors simultaneously with the vaccination, prior studies suggest that integration of adjuvant into the vaccine structure, if achievable, may result in higher innate immunity than adjuvant and vaccine mixing. As a result, adjuvants can be added to these vaccines as fused fragments. Recently, computer-aided vaccine development has been used as an innovative approach to developing various multi-epitope vaccinations [4].

Since multi-epitope vaccines are usually string-of-bead structures constructed from several separate segments joined together directly or through linkers, diverse constructs may be made by changing the order of the segments and using different linkers [5]. Linker usage is usually recommended in developing multi-epitope peptide vaccines to avoid the generation of junctional epitopes (neoepitopes) and promote the antigen presentation process. Multi-epitope polypeptide vaccines can be considered a type of fusion vaccination (albeit one with antigenic qualities), to which the fundamental principles for creating fusion proteins may apply [5–7]. When creating synthetic fusion proteins, several crucial considerations should be made, especially the sequence in which the fusing components are assembled, since this might impact the activity of the molecule’s domains and its general properties. The other important variable is the used linkers’ characteristics, including length, composition, and structure [7]. An essential factor in a vaccine’s effectiveness is its structural stability. The ideal structural stability of the vaccine construct is crucial for adequately presenting antigens, which can effectively trigger the immune response. Other physicochemical characteristics of the vaccine design, such as hydrophilicity-hydrophobicity, stability, and pI, may also be crucial to its creation or effectiveness [8].

The overuse of antibiotics has steadily resulted in the development of antibiotic and antifungal chemical resistance, creating several difficulties for public health and medicine [9]. The respiratory system’s nosocomial infections, including *Acinetobacter*, are among the worrisome developing bacteria [9, 10]. Among this species’ extracellular bacteria, *A. baumannii* is one of the most adept in developing antibiotic resistance, frequently failing therapeutic interventions [10]. Based on data from 18 studies, the overall pooled incidence of hospital-acquired *A. baumannii* infections (HA-AB) in the WHO regions of Europe, Eastern Mediterranean and Africa is 25.1 (95% CI 12.8–48.5) cases per 1000 patients [10–12]. Recently, specialized approaches or combinations of them, such as vaccination, monoclonal antibodies, and phage treatments, have been created to block many fundamental resistance mechanisms [13]. In light of this, immunization can effectively prevent this infectious agent, particularly in high-risk groups. As a Gram-negative, extracellular bacterial pathogen, *A. baumannii*, it is anticipated that B lymphocytes and immunoglobulin will be crucial in the host’s defense against infections [14].

Furthermore, outer membrane vesicles (OMV) and outer membrane compounds (OMC) have been used to create *A. baumannii* vaccines [14]. In essence, the epitopes on these proteins are appropriate for stimulating the immune system. The arrangement of these segments and the linker might affect the vaccine’s overall features [15].

There is no freely accessible tool for assessing and contrasting the many potential structures and their usefulness as a vaccine candidate, even though a commercial toolbox has been established for such research in vaccine design. Therefore, this work chose the optimal sequence among the developed structures based on a unique reverse vaccinology technique for comparing various synthesized molecules using computational approaches. Docking and molecular dynamics (MD) modeling of the developed constructs were also investigated, in addition to comparing their physicochemical characteristics, secondary and tertiary structures, and conformational B-cell epitopes [16]. Docking and molecular dynamics (MD) modeling of the developed constructs were also investigated, in addition to comparing their physicochemical characteristics, secondary and tertiary structures, and conformational B-cell epitopes. These systems are simple to use, aid in identifying potential vaccinations, and help save time and money.

## Material and methods

### Upstream in silico analyses

#### The identification of a possible vaccination candidate

For this investigation, 35 genome sequences of *A. baumannii* strains were found using the Vaxign database (http://www.violinet.org/vaxign/). Candidates for vaccines were selected during the 40 gene-coding protein investigations. The relevant parameters were used: no similarity to mammalian proteins, at least one transmembrane helix (≤1), and a frequency of adhesion higher than 0.51. Proteins from *A. baumannii* will be utilized for further study if the BLASTp website verifies the sensitivity of the protein. The *A. baumannii* proteome was examined utilizing the UNIPORT database to look for the existence of various proteins. The NCBI (http://www.ncbi.nlm.nih.gov/protein) then recorded the amino acid sequences of these proteins in FASTA format for future research.

#### Analyzing the immunostimulatory potential of *A. baumannii* proteins

##### Prediction of the virulent, antigenic, flexible, and hydrophilicity properties

The VirulentPred database (http://203.92.44.117/virulent/) was used to analyze the pathogenicity feature of possible vaccine candidates. In this study, the cascade SVM module with a threshold value of 0.5 (≥0.5) was used. A robust method for identifying antigenic properties of proteins is the VaxiJen database. Instead of sequence alignment, this server was used to characterize proteins’ antigenic properties purely based on their physicochemical properties. The VaxiJen algorithm (http://www.ddg-pharmfac.net/vaxijen/VaxiJen/VaxiJen.html) was used to calculate the antigenicity of the selected proteins, using a cut-off of 0.5. Utilizing http://tools.immuneepitope.org, the average flexibility and hydrophilicity of molecules were also determined.

##### Identify protein solubility and MHC binding locations

The level of solubility for a particular protein may be a good indicator of how well it functions. In vaccination projects, more than 30% of the produced molecules are not soluble [17]. The SOLpro (https://protein-sol.manchester.ac.uk/) program employs a two-step SVM approach to estimate protein solubility. As a result, SOLpro estimated the proteins’ solubility.

Utilizing Vaxitop (http://www.violinet.org/vaxign/vaxitop/), the number of Major Histocompatibility Complex class I (MHC I) and class II (MHC II) ligands (in mice) was evaluated (*P*-value ≤0.01). The proteins with the highest ratio (the cut-off was > 95% CI) were selected for further study.

### Downstream in silico analyses

#### Identifying protein-conserved domains

Utilizing Pfam 32.0 and the Conserved Domain Database, CDD (https://www.ncbi.nlm.nih.gov/Structure/cdd/cdd.shtml), the essential protein domains were identified. Protein sequences may be annotated with the location of conserved domains using CDD, a function of NCBI’s Entrez query. The vast majority of transmembrane proteins in Pfam 32.0 are characterized by many hidden Markov models and sequence alignments (HMMs).

Predicting the location of protein epitopes and their positioning surfaces is crucial for vaccine design. In order to identify protein sequences and their transmembrane localization, the PRED-TMBB (bioinformatics.biol.uoa.gr) was employed. In order to identify membrane segments, determine ring topology, and apply discriminatory algorithms on amino acid sequences, use this website.

#### Fusion vaccine design

Following significant immunoinformatic and computational research, 20 antigenic epitope sequences were chosen from the outer membrane proteome of *A. baumannii*. Various linkers (2HEYGAEALERAG linker + 17 GGGS linker) then connected these peptide fragments to create two different constructions. These two constructions’ basic structures were examined.

#### Structural analyses of vaccine

##### Examining physicochemical characteristics

Some of the physicochemical properties of the constructed structures that were assessed using the ProtParam platform (http://web.expasy.org/protparam/) included Instability index, grand mean of hydropathicity (Gravy), conceptual pI, the overall number of negative charges residues (Asp + Glu), and the overall number of positive charge residues (Arg + Lys).

The ccSOL omics website (http://service.tartaglialab.com/grant submission/ccsol omics) estimated the solubility of the various structures based on the protein’s tendencies for disorder, coil, and hydrophilicity.

##### The amino acids’ secondary structures and exposures

The structural features of the intended segments were projected by three renowned data centers: PORTER (http://distill.ucd.ie/porter/), SPIDER2 (http://sparkslab.org/server/SPIDER2/index.php), and PSIPRED v3.3 (http://bioinf.cs.ucl.ac.uk/psipred/). Two data centers: PaleAle (http://distill.ucd.ie/paleale/) and SPIDER2 (http://sparks-lab.org/server/SPIDER2/index.php), were used to estimate the explosibility or solvent accessibility (ACC) of TLR4 agonist in various constructed structures.

##### Modeling and optimization of proteins

The I-Tasser program, a study for automatic vehicle proteomics, was used to simulate the three-dimensional structure of the two proposed vaccine prototypes. This service goes through the following stages: A) Threading (finding template molecules from the PDB that have close similarity to the query proteome using a variety of connector alignment techniques); B) Systemic assembly (segment assembly that used an altered prototype Monte Carlo simulation technique in addition to ab initio modeling for some locations, essentially loops and tails); C) Model identification and precision (collection of the prototype by using cluster analysis structure decoys and precision by fragment).The I-Tasser defines a *value* known as the “C-score” as a probability value for calculating the model’s accuracy rate. Models with more probability have higher C-score values (which range from [5 to 2]). The Discovery Studio 3.5 and UCSF Chimera programs rendered the 3D modeled buildings.

##### Modeling of conformational B-cell epitopes

Immunogenicity was estimated utilizing IEDB databases on Kolaskar and Tonga Onkar antigenic levels. The highest, average, and lowest B-Cell epitope immunostimulatory scores were obtained using this dataset. The antigenic cutoff was set at 1.000. Complete proteins are generally categorized as possibly immunostimulatory on this platform if their score is more than 1.000.

Additionally, the discontinuous B-cell epitopes were identified using the DiscoTope 2.0 website (http://www.cbs.dtu.dk/services/DiscoTope/) after modeling the protein’s 3D structure. The resulting scores are calculated by this site using two distinct approaches: contact numbers generated from membrane availability and a unique epitope probability amino acid value. The standard threshold (− 3.7) was employed, and at that level, the sensitivity and specificity were both 0.47 and 0.75.

##### Molecular docking analyses: examination of the relationship between the TLR4 agonist and the receptor

Using the ClusPro website at https://cluspro.org/home.php, docking investigations were used to examine how the TLR4 agonist RS09 interacted with TLR4 in each molecule. Using the ClusPro website, rigid-body protein-protein interaction is carried out. Desolvation and electrically charged energies are used to select and rank attached structures with excellent surface compatibility, which is fully automated and quick. The local minima are smoothed using clustering, and the ones with the broadest potential wells—a characteristic linked to the limitless energy at the binding site—are chosen. Mouse TLR4 protein with ID number Q9QUK6 from the UniProt library was used to interact RS09 with TLR4. Additionally, PDB’s algorithm 3fxi was used to derive its 3D structure.

##### Assessment of immunogenicity and allergenicity

The ideal construct was evaluated for allergenicity and immunogenicity based on the findings of docking assays. The ANTIGENpro website (http://scratch.proteomics.ics.uci.edu/) and the VaxiJen v2.0 website (http://www.ddgpharmfac.net/vaxijen/VaxiJen/VaxiJen.html) performed the immunogenicity evaluation, respectively. Algpred web server at http://www.imtech.res.in/raghava/algpred/ conducted the allergenicity assessment.

##### Validation and improvement of the 3D modelled structure

The GalaxyRefine website at http://galaxy.seoklab.org/cgi-bin/submit.cgi?type=REFINE refined the chosen Structure. The model’s performance was then confirmed using the ProSA-web, ERRAT, and RAMPAGE (Ramachandran Plot Assessment) (http://mordred.bioc.cam.ac.uk/rapper/rampage.php) websites after the 3D improved structures had been evaluated. For the next round, the most modified model was chosen.

##### Molecular dynamics experiments

The chosen docking model was employed as the starting configuration in modeling molecular dynamics (MD) procedure to guarantee the bonding stability of the planned vaccination to TLR4/MD2 interaction. The TLR4/MD2-vaccine complex is given a reasonable chance to achieve optimal alignment and linkages concerning one another using MD modeling. GROMACS 5.0.1 was used to run a 30-ns MD modeling on the TLR4/MD2-vaccine compound.

##### Utilizing in-silico cloning and optimizing the developed vaccine’s codon

JCAT (http://www.jcat.de/) and Wrangler (https://www.mrc-lmb.cam.ac.uk/ms/methods/codon.html) server tools were used to optimize heterologous protein production in the mouse model. The RF-Cloning program employed the nucleotide sequence of the ultimate vaccine construct produced in vector pcDNA3.1 (+) (https://rf-cloning.org/) to assure vaccine production. Independent transcription terminators, prokaryotic ribosome binding sites, and multiple enzyme restriction sites were also developed.

## Results and discussion

### Upstream in silico analyses

#### Prediction of antigens

Thirteen antigens were discovered, and the ones with the most significant resemblance were selected. Protein screening was carried out following the instructions specified in the protocols. The BLASTp alignment results for the selected proteins are shown in Table 1. The table shows the most significant identification of the various Acinetobacter strains with the query strains and the query coverage. With localization scores > 2, eight of the 13 chosen proteins were predicted to be surface proteins. They were including 34 kDa outer membrane protein, Omp38, Omp W, CarO, putative porin, OmpA, FhuE, and CdiA. MlaA, OmpF and NlpB were eliminated from further study because they are periplasmic proteins.

**Table 1:** Preliminary protein detection and profiling

Protein	Symbol	Accession numbers	Cello protein		Vaxign			VaxiJen
			Region localized	Score	Adhesin Probability	Similarity to Host Proteins	Y/N	
34 kDa outer membrane protein	34 kDa	QEE55728.1	OuterMembrane	3.61	0.279	Human	N	0.77
						Mouse	N	
						Pig	N	
Omp38	Omp38	EKU52893.1	OuterMembrane	2.46	0.492	Human	N	0.76
						Mouse	N	
						Pig	N	
Omp W	Omp W	SST46813.1	OuterMembrane	2.07	0.291	Human	N	0.71
						Mouse	N	
						Pig	N	
ornithine uptake porin CarO	CarO	WP_163049344.1	OuterMembrane	3.16	0.688	Human	N	0.59
						Mouse	N	
						Pig	N	
putative porin	porin	OUM78068.1	OuterMembrane	3.61	0.854	Human	N	0.74
						Mouse	N	
						Pig	N	
OmpA	OmpA	AXV53527.1	OuterMembrane	3.07	0.629	Human	N	0.81
						Mouse	N	
						Pig	N	
contact-dependent growth inhibition A	CdiA	QDC12537	OuterMembrane	2.4	0.310	Human	N	0.57
						Mouse	N	
						Pig	N	
Fe(III)-rhodotrulic acid uptake	FhuE	ABO12504	OuterMembrane	2.6	0.310	Human	N	0.53
						Mouse	N	
						Pig	N	
putative phospholipid-binding lipoprotein MlaA	MlaA	ATP88409	Periplasmic	3.5	0.146	Human	N	0.53
						Mouse	N	
						Pig	N	
Outer membrane protein	OmpF	CDG80059	Periplasmic	2.21	0.510	Human	N	0.77
						Mouse	N	
						Pig	N	
outer membrane *protein* assembly complex	NlpB	SUU39799	Periplasmic	2.68	0.42	Human	N	0.62
						Mouse	N	
						Pig	N	
Lactate utilization protein A	LutA	SVK36206	Cytoplasmic	7.9	0.125	Human	N	0.52
						Mouse	N	
						Pig	N	
AdeABC efflux pump	AdeS	ADM92606	InnerMembrane	10	0.283	Human	N	0.51
						Mouse	N	
						Pig	N	

LutA (As cytoplasmic proteins) and AdeS (as internal membrane proteins) were disregarded from further investigation. The eight outer membrane proteins were selected and moved on to the subsequent investigation phase based on the Cello protein analysis results (Table 1). The Vaxign web server evaluated the similarities to proteins identified in humans, mice, and pigs. This program allows users to dynamically input protein sequences and choose parameters as vaccination targets. The results of these evaluations are shown in Table 1. Also, the similarity to the human genome was evaluated using BLASTp. Table 1 shows that no protein significantly resembled *Homo sapiens*.

The antigenicity was calculated using the VaxiJen server with a cut-off size of ≥0.5. As can be seen in Table 1, the proteins 34 kDa outer membrane protein, Omp38, Omp W, CarO, putative porin, OmpA, were chosen as having the right antigenicity (≥0.5). FhuE, and CdiA were eliminated from further study because their low antigenicity. Other proteins were eliminated due to their periplasmic/cytoplasmic nature. The eight proteins were used as suitable proteins to assess solubility and flexibility.

#### Protein solubility prediction

**Fig. 1:**
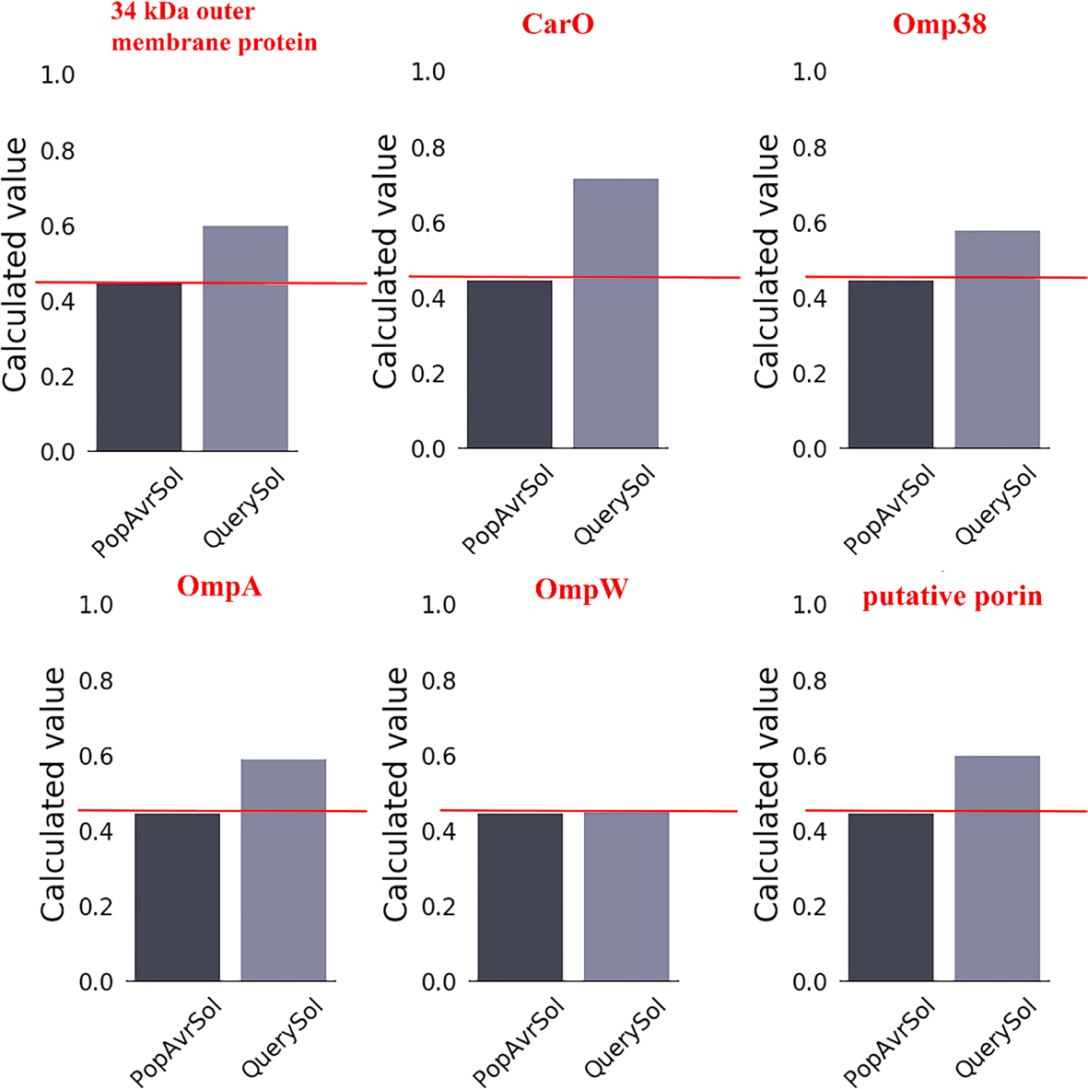
Protein solubility diagram. According to the results, the proteins showed more solubility than the control (PopAvrSol). However, the ompW protein showed the same solubility as the control (PopAvrSol)

The amount of a protein’s function may be determined by its solubility. With 78% accuracy, soluble and insoluble proteins produced by heterologous expression studies may be distinguished using information about a protein’s solubility. Figure 1 shows that 5 of the six examined proteins (34 kDa outer membrane protein, Omp38, CarO, putative porin, OmpA) exhibited suitable solubility ranges. Omp W, however, lacked the proper solubility ranges. The SolPro server was used to calculate solubility. Proteins with values lower than 0.45 were deemed insoluble, whereas those with scores greater than 0.45 were deemed soluble. Therefore, 34 kDa outer membrane protein, Omp38, CarO, putative porin, and OmpA scored 0.63, 0.59, 0.78, 0.61, and 0.59, respectively, demonstrating adequate solubility. Additionally, the solubility of ompW was 0.45.

**Fig. 2:**
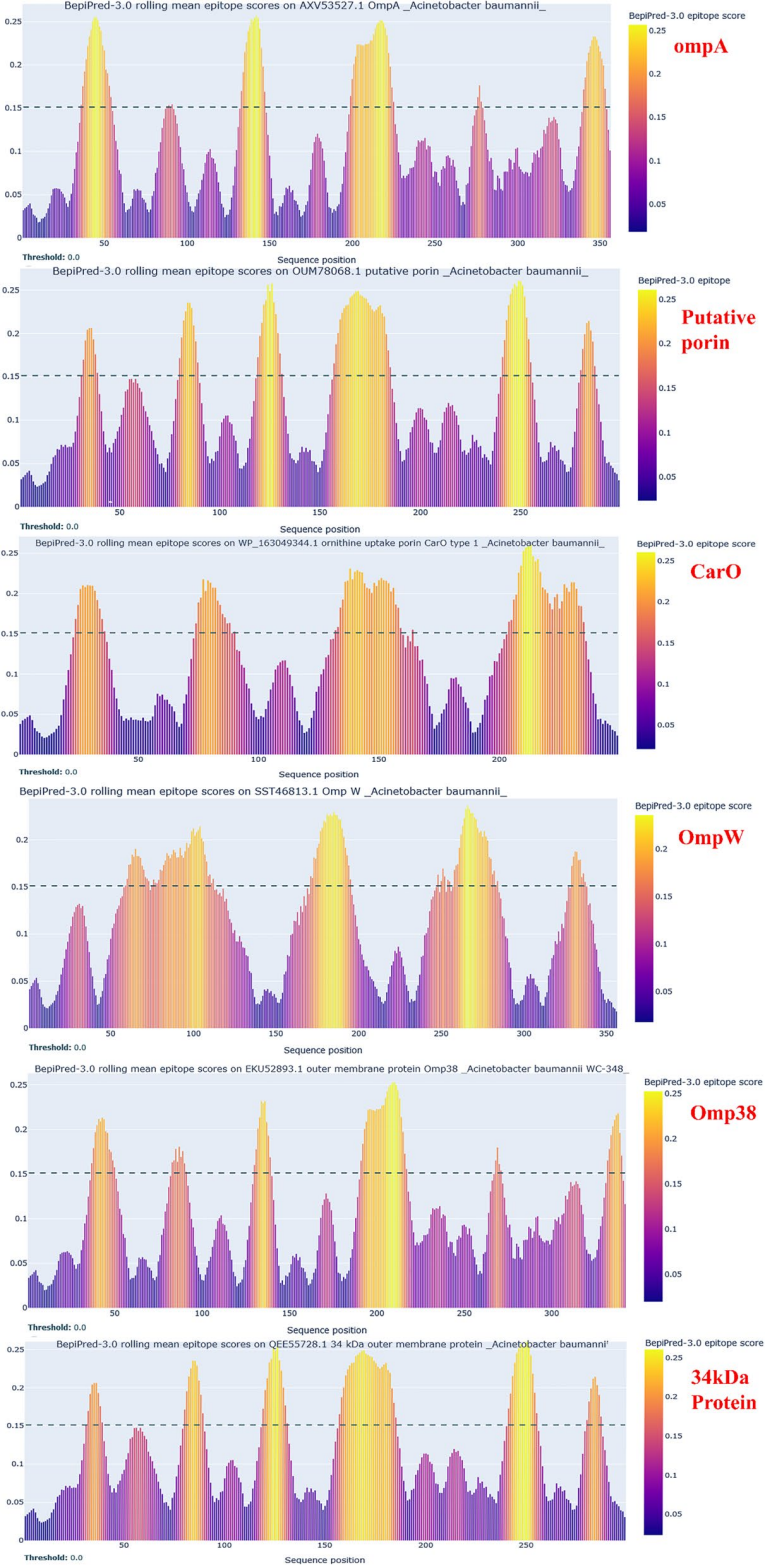
BepiPred-2.0 dataset for linear B cell epitopes (The BepiPred-2.0 and NetSurfP predictions for each query sequence are shown on the Summary output page in Advanced Output mode). The sequence of the orange gradient protein displays positions above the threshold epitope. As a result, all proteins that passed the screening and had an orange coloration of at least 70% had robust epitope features

The 34 kDa outer membrane protein, Omp38, OmpW, CarO, and putative porin, OmpA, were examined for their suitability for antigenicity and immunogenicity. BepiPred-2.0 was trained on linear epitopes. Thus, the program’s effectiveness was evaluated using a dataset of confirmed positive and negative peptides obtained from the immune epitope dataset to guarantee a fair comparison. The results of this benchmark are shown in Fig. 2. Using NetsurfP, Helix (H - pink probability gradient), Sheet (E - blue probability gradient), and Coil (C - orange probability gradient) were predicted. Surface: Predicted relative surface accessibility shown as Buried(B)/Exposed(E) using NetsurfP’s default threshold and an orange gradient. Positions over the threshold epitope are shown in the orange gradient protein sequence. As a result, all proteins tested with an orange coloration of more than 70% had robust epitope features of sequences. The 34 kDa outer membrane protein, Omp38, OmpW, CarO, and putative porin, OmpA with an average score of > 0.1512, was determined to be the acceptable antigenic candidate.

### Downstream in silico analyses

#### Identification of external epitopes and epitope localization

**Fig. 3:**
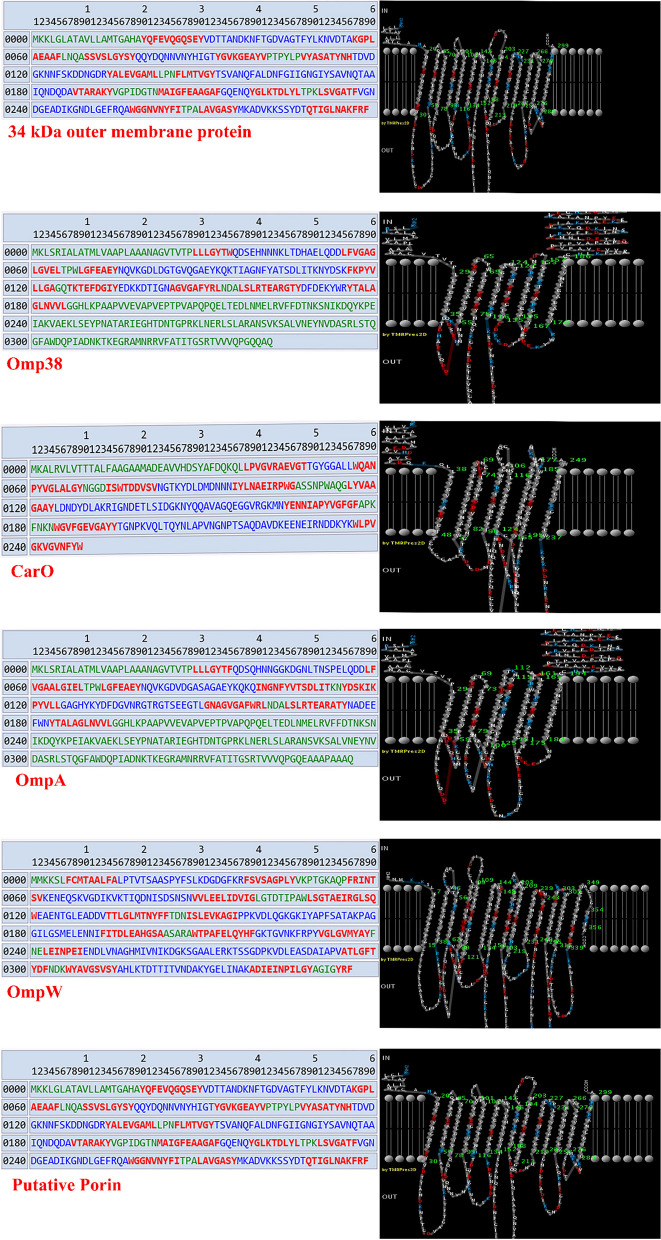
Localization of proteins epitopes in the bacterial cell membrane. Blue sequences are located on the outer membrane, red sequences on the periplasmic space, and green sequences on the inner membrane. Also, the image on the right shows the 2D representation

According to the findings of Fig. 3, PRED-TMBB indicated the weight of certain protein epitopes in the bacterial cell membrane. Sections of the proteins inside the inner membrane were colored green, the parts across the membrane were red, and the portions outside of the membrane or on the surface of the bacterial membrane were colored blue. Following the findings, each of the chosen proteins had its unique membrane weight, as shown in Fig. 3.

The epitope sequences of the chosen proteins were acquired from IEDB, as can be shown in Table 2. Using the starting and ending amino acid values of the epitope, the sequence was superimposed over the PRED-TMBB findings, and outer and inner membrane sections were detected and then verified as functional areas of each protein. If the epitopes are more in the outer layer and the amino acid sequences in the outer layer are longer, the immunogenicity of the obtained protein will be higher. According to this study, all 6 proteins—34 kDa outer membrane protein, Omp38, OmpW, CarO, and putative porin, OmpA—scored much higher than the cutoff point. Table 2 exactly shows the antigenic property of the analyzed protein. Each of the proteins 34 kDa outer membrane protein, Omp38, OmpW, CarO, and putative porin, OmpA were identified with average antigenicity as they contained some epitopes projecting from the membrane surface, as shown in Table 2.

**Table 2:** Comparison of the selected proteins with the PRED-TMBB database (http://bioinformatics.biol.uoa.gr/PRED-TMBB/) to evaluate extracellular epitopes

Protein	Epitope		Epitope Type	Out	Epitope outer part
	Start- End	Initial Epitope Sequence			
**34 kDa outer membrane protein**	78–85	**YQQYDQNN**	B-cell	78–85	**YQQYDQNN**
	111–134	**SATYNHTDVDGKNNFSKDDNGDRY**		116–133	**TDVDGKNNFSKDDNGDR**
	179–190	**AAIQNDQDAVTA**		179–187	**AAIQNDQDA**
	211–220	**GAFGQENQYG**		215–218	**QENQ**
	237–253	**FVGNDGEADIKGNDLGE**		237–253	**FVGNDGEADIKGNDLGE**
	281–290	**VKKSSYDTQT**		281–288	**VKKSSYDT**
	31–39	**VPTPYLPVY**	MHC-I/MHCII combined coverage	31–39	**VPTPYLPVY**
	40–49	**FTGDVAGTFY**		40–49	**FTGDVAGTFY**
	47–55	**AWGGNVNYF**		47–55	**AWGGNVNYF**
**Omp38**	35–46	**WQDSEHNNNKLT**	B-cell	35–46	**WQDSEHNNNKLT**
	74–96	**EYNQVKGDLDGTGVQGAEYKQKT**		74–96	**EYNQVKGDLDGTGVQGAEYKQKT**
	125–145	**GQTKTEFDGIYEDKKDTIGNA**		136–144	**EDKKDTIGN**
	34–42	**TSDLITKNY**	MHC-I/MHCII combined coverage	36–42	**DLITKNY**
	43–51	**DSKFKPYVL**		43–51	**DSKFKPYVL**
	49–57	**HLKPAAPVV**		49–56	**HLKPAAPV**
**OmpW**	19–24	**VTSAAS**	B-cell	19–24	**VTSAAS**
	27–35	**FSLKDGDGF**		27–35	**FSLKDGDGF**
	61–71	**SVKENEQSKVG**		63–71	**KENEQSKVG**
	80–87	**DNISDSNS**		80–87	**DNISDSNS**
	120–133	**QWEAENTGLEADDV**		122–133	**EAENTGLEADDV**
	154–165	**AGIPPKVDLQGK**		156–165	**IPPKVDLQGK**
	265–292	**DGKSGAALERKTSSGDPKVDLEASDAIA**		265–292	**DGKSGAALERKTSSGDPKVDLEASDAIA**
	173–181	**SATAKPAGG**		173–181	**SATAKPAGG**
	323–330	**TITVNDAK**		323–330	**TITVNDAK**
	19–27	**FTYDFNDKW**	MHC-I/MHCII combined coverage	19–27	**FTYDFNDKW**
	61–69	**GSAASARAW**		63–69	**AASARAW**
	31–39	**GSVSYAHLK**		31–37	**GSVSYAH**
**CarO**	44–51	**AEVGTTGY**	B-cell	44–51	**AEVGTTGY**
	70–91	**NGGDISWTDDVSVNGTKYDLDM**		82–91	**VNGTKYDLDM**
	134–170	**RIGNDETLSIDGKNYQQAVAGQEGGVRGKMNYENNIA**		134–170	**RIGNDETLSIDGKNYQQAVAGQEGGVRGKMNYENNIA**
	193–202	**AYYTGNPKVQ**		196–202	**TGNPKVQ**
	208–235	**LAPVNGNPTSAQDAVDKEENEIRNDDKY**		208–235	**LAPVNGNPTSAQDAVDKEENEIRNDDKY**
	49–57	**TGYGGALLW**	MHC-I/MHCII combined coverage	49–56	**TGYGGALL**
**OmpA**	36–56	**QDSQHNNGGKDGNLTNSPELQ**	B-cell	36–56	**QDSQHNNGGKDGNLTNSPELQ**
	78–100	**EYNQVKGDVDGASAGAEYKQKQI**		78–100	**NQVKGDVDGASAGAEYKQKQ**
	134–153	**FDGVNRGTRGTSEEGTLGNA**		134–153	**FDGVNRGTRGTSEEGTL**
	172–181	**RATYNADEEF**		172–181	**YNADEEF**
	37–45	**TSDLITKNY**	MHC-I/MHCII combined coverage	37–45	**TSDLITKNY**
	46–54	**DSKIKPYVL**		46–54	**DSKIKPYVL**
	50–59	**RVFATITGSR**		50–57	**RVFATITG**
**putative porin**	24–43	**VQGQSEYVDTTANDKNFTGD**	B-cell	31–43	**VDTTANDKNFTGD**
	78–85	**YQQYDQNN**		78–85	**YQQYDQNN**
	111–134	**SATYNHTDVDGKNNFSKDDNGDRY**		116–133	**TDVDGKNNFSKDDNGDR**
	179–180	**AAIQNDQDAVTA**		179–188	**AAIQNDQDAV**
	211–220	**GAFGQENQYG**		214–218	**GQENQ**
	237–253	**FVGNDGEADIKGNDLGE**		237–253	**FVGNDGEADIKGNDLGE**
	281–290	**VKKSSYDTQT**		281–288	**VKKSSYDT**
	31–39	**VPTPYLPVY**	MHC-I/MHCII combined coverage	31–39	**VPTPYLPVY**
	40–49	**FTGDVAGTFY**		40–49	**FTGDVAGTFY**
	46–54	**QAWGGNVNY**		46–54	**QAWGGNVNY**

#### The vaccine construct is being developed

The vaccine design was developed by combining the most effective B-cell, and MHC-I/MHCII combined coverage epitopes with the help of HEYGAEALERAG and GGGS linkers. The EAAAK linker was used to connect the adjuvant CPG ODN 2395 (5′-cgtcgttttcggcgcgcgccg-3′) to the front of the vaccine. In the current experiment, 10 B-cell epitopes, and 10 MHC-I/MHCII combined coverage epitopes were chosen from the *A. baumannii* structural protein sequences, respectivly. We created a vaccine design after careful combination and randomization. The final vaccine construct was shown in Table 3. Also, gene constructs Omp38 + omp W + CarO and Omp38 + putative porin + omp A were designed, which were rejected in the initial analysis, such as the immunogenicity score, solubility, and interaction with other proteins. Therefore, these structures were not used in the subsequent studies.

**Table 3:** The physicochemical properties of the generated sequence of vaccine structures designed in this study

Epitope composition	group	Vaccine sequence	AlgPred	ccSOL (< 0.4)	PI	GRAVY	VaxiJen	homology
**Multi epitope for** ***A. baumannii***	Adjuvant+ 10 B-cell epitope+ 10 MHC-I/MHCII combined coverage epitope +Adjuvant	MSPSVRHSPSVRHEAAAKVPTPYLPVYHEYGAEALERAGFVGNDGEADIKGNDLGEGGGSYQQYDQNNGGGSTSDLITKNYGGGSEYNQVKGDLDGTGVQGAEYKQKTGGGSWQDSEHNNNKLTGGGSFTYDFNDKWGGGSGSAASARAWGGGSDGKSGAALERKTSSGDPKVDLEASDAIAGGGSSATAKPAGGGGGSAWGGNVNYFGGGSTGYGGALLWGGGSRIGNDETLSIDGKNYQQAVAGQEGGVRGKMNYENNIAGGGSAEVGTTGYGGGSDSKFKPYVLGGGSFTGDVAGTFYGGGSFVGNDGEADIKGNDLGEGGGSTSDLITKNYGGGSDSKIKPYVLHEYGAEALERAGQDSQHNNGGKDGNLTNSPELQEAAAK	NON ALLERGEN	0.641	4.58	−0.618	1.51	non-homologue

#### Antigenicity, Allergenicity, and physicochemical profile of vaccines

Ten B-cell epitopes, and 10 MHC-I/MHCII combined coverage epitopes were chosen from the *A. baumannii* structural protein sequences, respectively. The physicochemical properties of the generated component were evaluated using the Expasy website. The molecular formula of vaccine was determined to be C_1718_H_2615_N_507_O_630_S_17_. The vaccine form has a molecular weight of 40,996.70 Da (suggesting a mean mass) and 47 negatively charged residues (Asp + Glu), whereas 28 positively charged residues (Arg + Lys). The estimated half-life were 7.2 hours (mammalian reticulocytes, in vitro), > 20 hours (*yeast*, in vivo) and > 10 hours (*Escherichia coli*, in vivo) for vaccine. The predicted PI characteristic of 4.58 showed that the vaccination was alkaline for vaccine. Furthermore, the aliphatic index value of vaccine was 43.14, and the instability value obtained was 24.30, This classifies the protein as stable. Given the value of the grand mean of hydropathicity (vaccine: − 0.618), the structure’s character was hydrophilic (GRAVY). The allergenicity and antigenicity of the vaccines were created. The antigenicity scores for vaccine were determined to be 1.51 on the Vaxijen2.0 server. Notably, the vaccinations were shown to be antigenic and non-allergenic across all servers. Moreover, the Protein-Sol server indicated that the vaccine design was soluble, with scores of 0.641 (Table 3). Furthermore, no TM helices or signal peptides were found in the vaccine constructs’ respective prediction findings.

#### BLAST homology analysis

The sequence homology between the produced vaccination protein sequence and the human proteome sequence indicated that the vaccine construct’s query coverage had not similarity (0%) to *Homo Sapiens* (Taxid:9606) proteins (Table 3). The BLAST homology evaluation result revealed that the projected vaccination protein would not trigger autoimmune reactions in the host.

#### The multi-epitope vaccine adopts a favored 3D structure

Structure templates found in the PDB collection are used by the I-TASSER service to begin modeling. The server may produce tens of thousands of pattern alignments, but it only employs those that have the greatest significance as determined by the Z-score (> 1 signify excellent alignment), which is a measure of significance. Five probable tertiary forms of the multi-epitope vaccine were predicted using the 10 best precursors (Z-score range: 1.01 to 5.59). All five models each had a unique C-score value: − 0.41, − 2.34, − 2.59, − 4.30, and − 2.70. The average C-score range is between − 5 and 2, with scores > − 1.5 suggesting a proper global topology. So, for our multi-epitope three-dimensional structure, we choose the C-score − 0.41 model (MODEL 1). The finest 3D model was selected, and the GalaxyWeb service then underwent a refining process to enhance the structural quality (https://galaxy.seoklab.org/cgi-bin/report_REFINE.cgi?key=93ba2a21a45fb1286116331ec3f9e4eb). As a consequence, the server produced five improved models (Table 4).

**Table 4:** Models after refinement using Galaxy Refine server

Model	GDT-HA	RMSD	MolProbity	Clash score	Poor rotamers	Rama favored
Initial	1.000	0.000	1.951	14.4	0.0	95.8
MODEL 1	0.9808	0.516	1.543	10.6	0.0	99.0
MODEL 2	0.9411	0.978	1.700	6.8	1.1	95.8
MODEL 3	0.9397	0.978	1.676	5.0	0.0	93.8
MODEL 4	0.9538	0.839	1.924	11.2	0.0	94.8
MODEL 5	0.9500	0.866	1.899	8.1	0.0	92.7

**Fig. 4:**
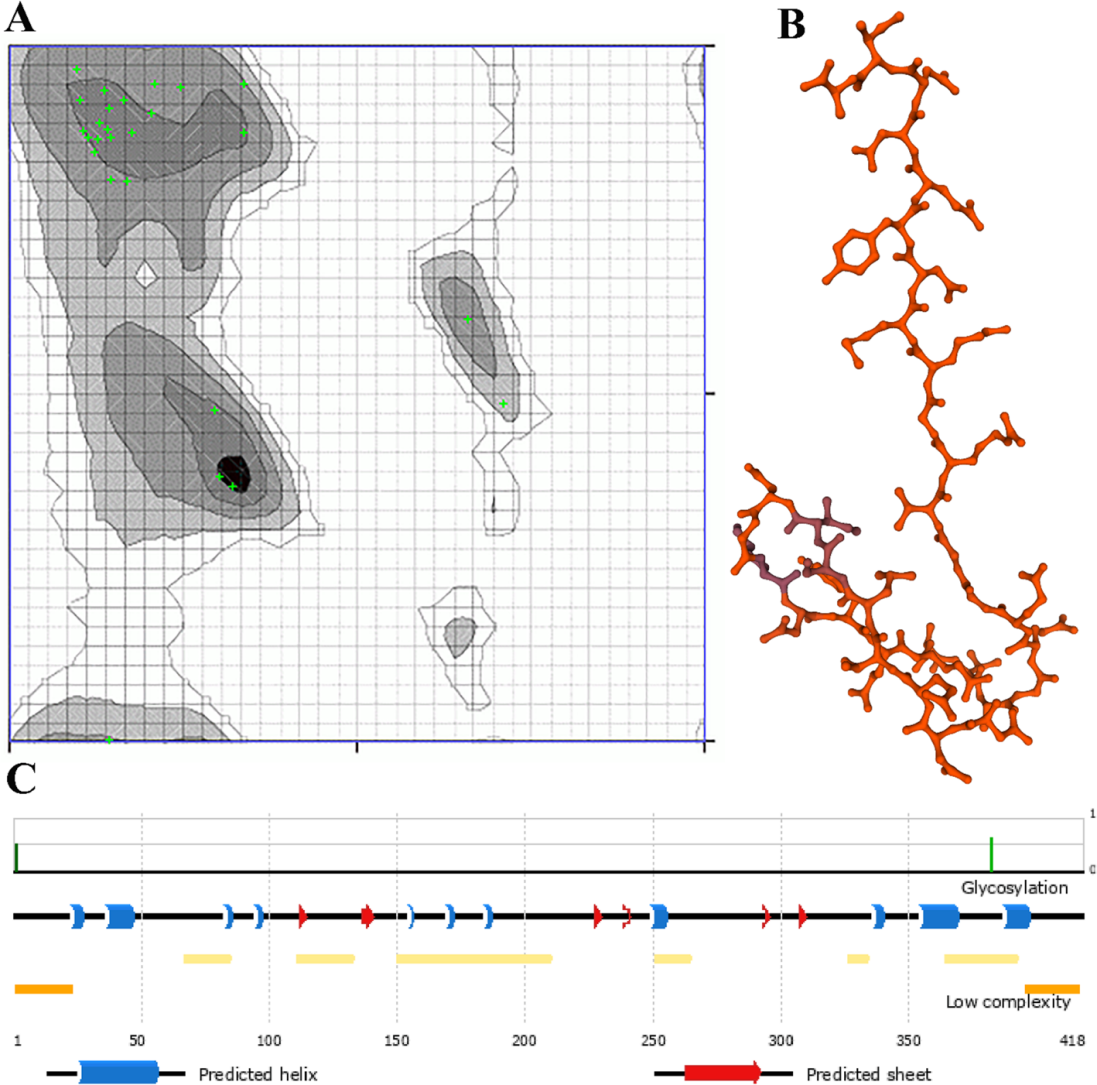
**A** Ramachandran Plot for vaccine. The chart is color-coded for your convenience: Black Dark Gray Gray Light Gray represent Highly Preferred Conformations (Delta > = − 2); White with Black Grid represents preferred conformations (− 2 > Delta > = − 4); White with Gray Grid represents questionable conformations (Delta < − 4); Highly Preferred observations shown as GREEN Crosses: 27 (100.000%). Preferred observations shown as BROWN Triangles: 0 (0.000%); Questionable observations shown as RED Circles: 0 (0.000%). **B** 3D models of vaccine construct. **C** Position dependent feature predictions are mapped onto the sequence schematic. The line height of the Phosphorylation and Glycosylation features reflects the confidence of the residue prediction

We evaluated the original and improved 3D models while taking into account the RAMPAGE server-generated Ramachandran plot to choose the best one. The analysis showed that total of residues was 27. The analysis showed that the original model placed 100% of the residues in preferred areas, 0% in permitted regions, and 0% in rejected regions (Fig. 4A). The model, included 27, 0, and 0% of the residues in preferred, permitted, and banned areas, respectively, after the refinement (Fig. 4A). 3D models of vaccine construct were shown in Fig. 4B. Also position dependent feature predictions are mapped onto the schematic sequence shown below (Fig. 4C). The line height of the Phosphorylation and Glycosylation features reflects the confidence of the residue prediction. The Biological Process Predictions result using FFPred Predictions was shown in Table S1. Global features are calculated directly from sequence. Localization values are predicted by the Psort algorithm and reflect the relative likelihood of the protein occupying different cellular localizations. The bias column is highlighted according to the significance of the feature value calculated from Z score of the feature (Table S2).

**Fig. 5:**
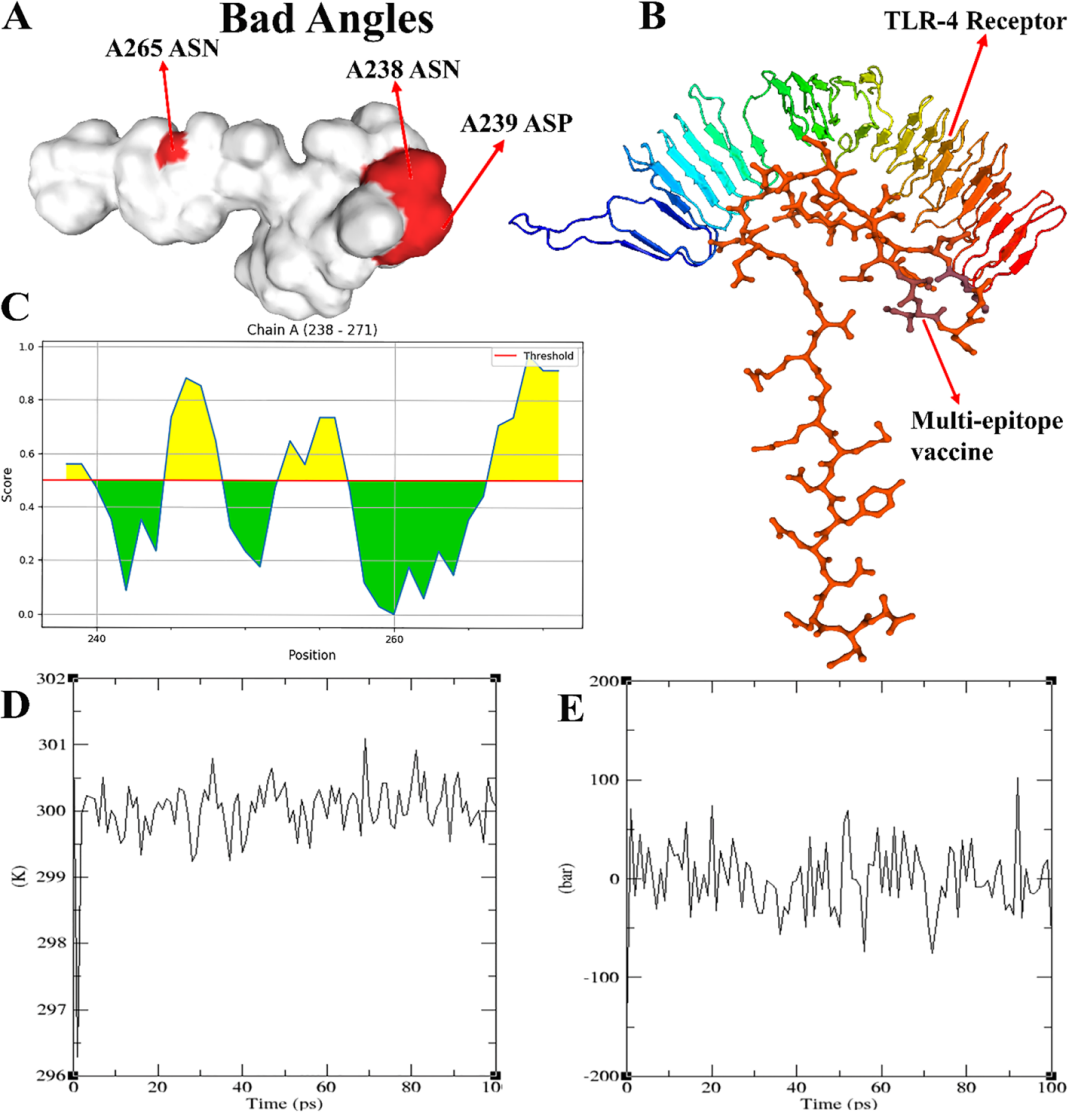
**A** MolProbity Results of Ramachandran plot Bad Angeles. **B** Ligand-receptor docked complex using the Swarmdock server. Docked structure visualization generated by Chimera software; TLR 4 (receptor) in top and the multi-epitope protein (ligand) in bottom. **C** The 2D Score Chart(s) of chain A B-cell epitope with ratios ranging 0.779 and 0.603. **D** The ligand-receptor complex was simulated using molecular dynamics. Changes in the ligand-receptor complex’s temperature (in Kelvin) when it is in the equilibration phase (100 ps). **E** The ligand-receptor pressure curve, calculated during the acclimatization period (100 ps)

MolProbity Results of Ramachandran plot show that MolProbity Score, Ramachandran Favoured, Clash Score and Ramachandran Outliers were 1.54, 100%, 0.00 and 0.00%. Also, Rotamer Outliers was 23.08% (A253 VAL, A270 ILE, A256 GLN, A257 GLU, A242 LEU, A261 ARG). C-Beta Deviations was 1 (A239 ASP) (Table S3). This model didn’t have Bad Bonds (0 / 259) but there was 3 Bad Angles (3 / 347) in A238 ASN, A265 ASN, A239 ASP according to Fig. 5A.

#### The multi-epitope vaccine interacts with the TLR 4 receptor

Our simulation and analysis of potential interactions between the ligand (multi-epitope vaccination) and the immunological receptor (TLR 4) were made feasible using the SwarmDock server and LIGPROT v2.2 software. The best-docked structure was chosen using the 43.71 minimal energy value. As seen in Fig. 5B, this complex displayed 21 hydrophobic contacts for the receptor, 29 hydrophobic interactions for the ligand, and 14 hydrogen bonds. These findings imply that our chimeric vaccination may trigger a crucial immune receptor, which is why a dynamic simulation was also carried out.

#### Folding of the putative vaccine produces conformational B-cell epitopes

**Fig. 6:**
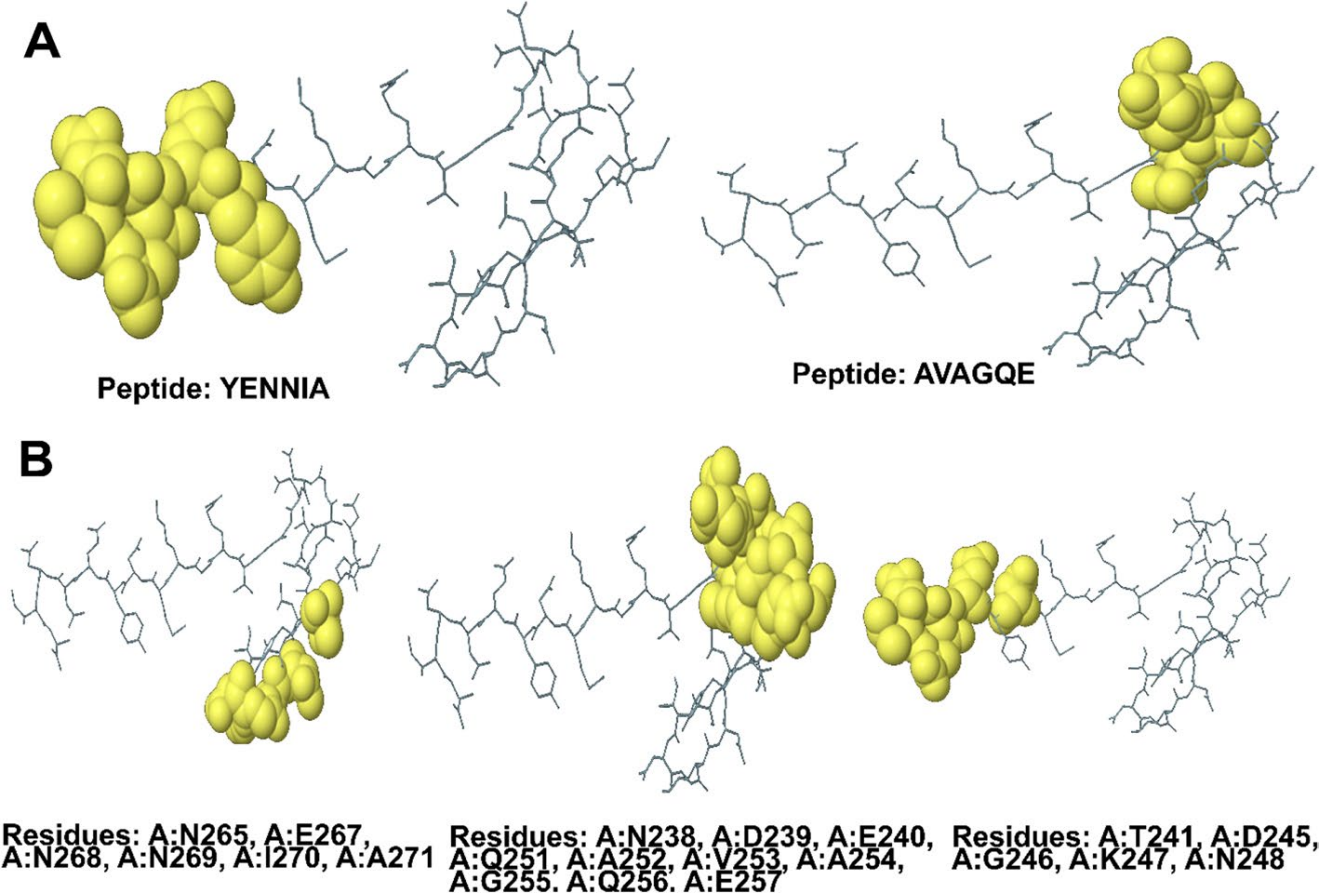
3D structure of (**A**) Predicted Linear Epitope(s) and (**B**) Predicted Discontinuous Epitope(s)

A total of 2 Linear B-cell epitopes with ratios ranging 0.779 and 0.603, and 3 Discontinuous Epitope with ratios ranging from 0.765, 0.694 and 0.538, included 21 residues, according to the ElliPro server (Tables 5 and 6). The 2D Score Chart(s) was shown in Fig. 5C. Also 3D structure of Predicted Linear Epitope(s) and Predicted Discontinuous Epitope(s) was shown is Fig. 6.

**Table 5:** Predicted Linear Epitope(s) for *A. baumannii*

No.	Chain	Start	End	Peptide	Number of residues	Score
**1**	A	266	271	YENNIA	6	0.779
**2**	A	252	257	AVAGQE	6	0.603

**Table 6:** Predicted Discontinuous Epitope(s) for *A. baumannii*

No.	Residues	Number of residues	Score
**1**	A:N265, A:E267, A:N268, A:N269, A:I270, A:A271	6	0.765
2	A:T241, A:D245, A:G246, A:K247, A:N248	5	0.694
3	A:N238, A:D239, A:E240, A:Q251, A:A252, A:V253, A:A254, A:G255, A:Q256, A:E257	10	0.538

#### Molecular dynamics foretells a stable binding agreement

An MD simulation was carried out to verify that TLR 4 was correctly activated when exposed to the potential multi-epitope vaccination. Following the energy minimization stage, the temperature and pressure equilibration phase was evaluated over 100 ps. During the period being studied, the temperature soon increased to 300 K (Fig. 5D). The pressure plot similarly showed that throughout the whole equilibration period, pressure fluctuated by around 0.25 bar (Fig. 5E). The completed trajectory was then examined to determine a few crucial metrics, the first of which was the RMSD. This value evaluates the stability between the receptor and ligand, where small changes indicate a steady contact. As a consequence, a 20-ns RMSD plot was produced, which is thought to show a minor variation of 0.25 to 1.5 nm. The second parameter, RMSF, indicates changes in amino acid side chains. The plot’s elevated fluctuations show extremely flexible areas in the receptor-ligand complex, while the plot’s moderate variations show ongoing interaction between receptor and ligand molecules. Except for one very flexible area (1.8 nm), the majority of our chosen complex has shown unbroken interactions (0.5 nm).

#### In silico cloning

**Fig. 7:**
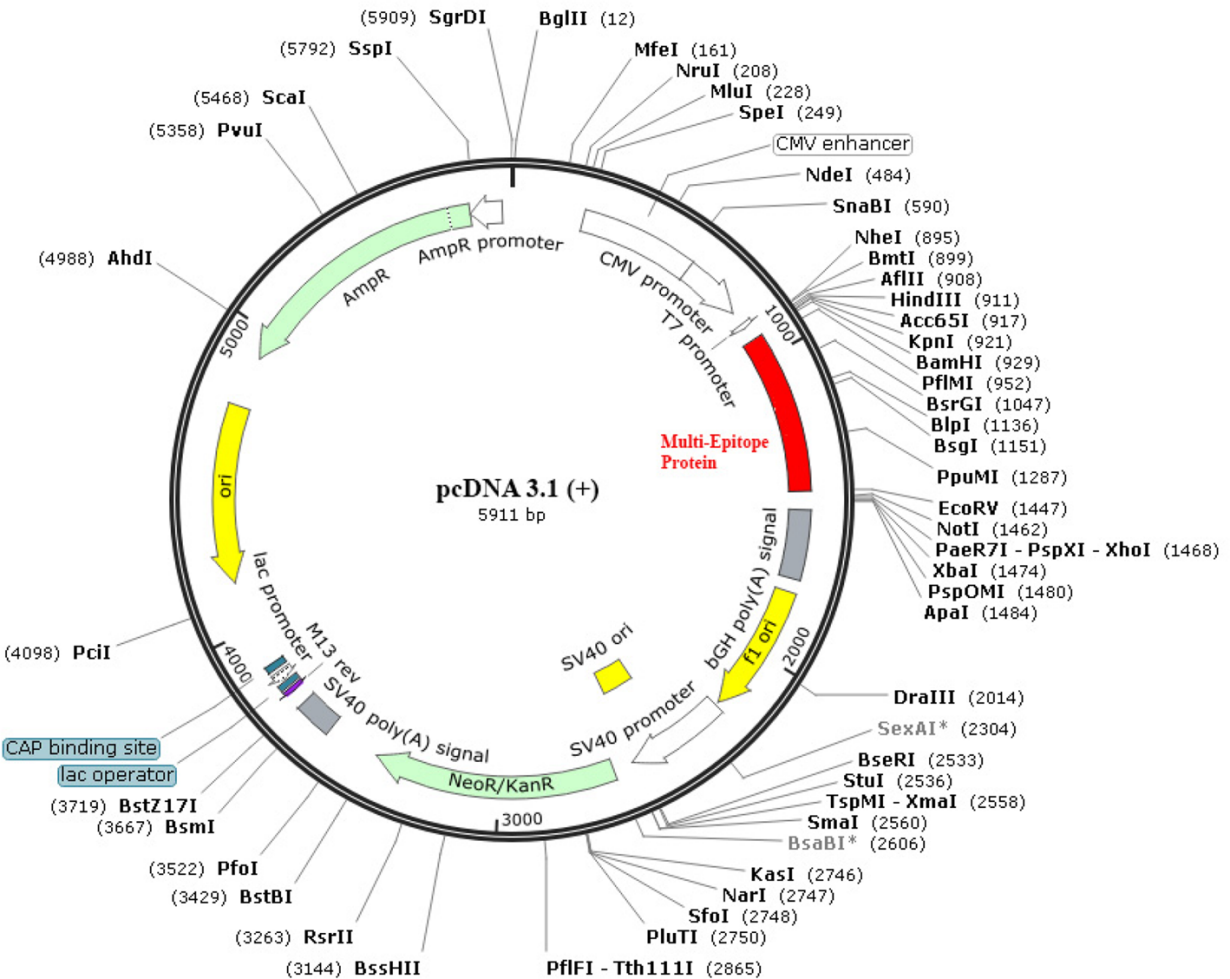
shows in silico cloning. A multi-epitope vaccination sequence that was cloned into the pET28a (+) expression vector is shown in red. The insert was inserted between the SalI and BamHI restriction sites

An optimized DNA sequence with excellent parameters was produced by the Jcat service. Indicating a significant likelihood of enhanced expression, the CAI score was 1.0. The amount of GC, which was at 54.25%, is still within the ideal range (30–70%). The multi-epitope vaccine insertion is carried via the expression vector pcDNA3.1 (+), which is shown in Fig. 7.

## Discussion

In the past, creating vaccines has been considered the most efficient way to treat illnesses brought on by infectious viruses [18]. Developing an effective antigen and suitable vaccine formulation against *A. baumannii* has taken decades. This endeavor enabled the discovery of numerous potential antigens and the formation of a shared understanding of the significance of vaccination in disease-fighting initiatives [18, 19]. Numerous strategies have been discussed, ranging from DNA vaccines to attenuated parasites, recombinant molecules, synthetic peptides, and chimeric proteins [20].

Given the capacity to combine immunogenic molecules in a single structure, chimeric or multivalent antigens, in particular constitute an alluring option. The first research on this strategy concerning *A. baumannii* was published over 20 years ago [21]. Since then, the creation of chimeric antigens has improved because of advancements in technologies like genomics and bioinformatics. The development of computer analysis has opened up opportunities such as screening of epitopes considering a wide variety of HLAs, structural analysis of the vaccine candidate, predictions of antigenicity, and assessment of interactions with human receptors [22].

Due to its efficiency in terms of time and money as well as safety, this immunoinformatic technique is particularly beneficial. As a result, several researchers have recently used similar techniques to study creatures, including viruses, bacteria, and parasites, producing intriguing compounds [23]. Using a multi-epitope strategy and a reliable in silico analysis, researchers recently announced the development of a promising vaccine candidate against various infections [17]. The seven main disease-related vaccination targets (Sm14, Sm21.7, Sm23, Sm29, Smp80, Sm-CB, and SM-TSP-2) that include T and B-cell epitopes were chosen by the authors as components of this potential antigen [24].

Here, we suggest creating a new multi-epitope vaccine candidate while considering a different protein source using a comparable approach. Because the surface proteins of pathogens are inherently likely to interact with the immune system of the host, they may trigger an immunological response [25]. Most exposed proteins are in an outer membrane layer in multicellular organisms like *A. baumanni*. We began our method by assessing all putative transmembrane sequences, 13 antigens, offered by the GeneDB database since there is not a database that specifically offers outer membrane protein sequences and because *A. baumannii* proteins may have potential that has yet to be fully explored. After that, filtering plasma membrane proteins was made possible by using PSORT II and CCTOP servers [26–28].

The physicochemical characteristics anticipated for the multi-epitope candidate greatly support the viability of heterologous expression and antigen purification as procedures. The use of this organism as a platform for heterologous expression has been suggested by the anticipated half-life in *E. coli* (10 h) and the stability of the molecules (aliphatic index 86.06). Based on *E. coli* strain K12, we have optimized vaccine candidate codons and carried out the in-silico cloning in pcDNA3.1 (+), a widely used expression vector. Additionally, the protein will adopt a negative global charge under neutral pH circumstances, according to the expected isoelectric point (5.93), which tends to facilitate affinity purification using columns with nickel (Ni2+) immobilized nickel [29, 30]. Furthermore, at these pH settings, significant changes in the protein structure are not anticipated to take place. Also supporting a successful manufacturing process is the solubility prediction (0.9010). Few compounds have reached clinical trials after decades of trying to find a viable antigen to serve as a suitable vaccine against *A. baumanni*, keeping the hunt for other methods open [31, 32].

The conservation of pathogen species, which may encourage cross-protection, is an appealing trait for vaccine antigens. The final proteins chosen for our analysis had a high degree of similarity with *A. baumanni* in terms of the epitopes they supplied for the chimeric putative antigen. On the other hand, because we have decided to deal with outer membrane proteins, there is no function accessible to them. However, when submitted to InterProScan, two final six proteins showed recognized superfamily motifs. Extracellular protein domains are often a significant source of vaccination candidates for several diseases. Similar to how studies of hypothetical proteins might lead to the identification of prospective candidates and encourage further research/annotation into them. As a result, we integrated these ideas to create a multi-epitope protein that demonstrated its suitability as a potential antigen. Our vaccine candidate may show encouraging results in upcoming in vitro and in vivo experiments according to immunogenic, physicochemical, and structural features.

### Supplementary Information

**Additional file 1.** The information is presented in online version.

## Data Availability

The datasets analyzed during the current study are available from the corresponding author upon reasonable request.

## Abbreviations

OMP: Outer membrane protein
APC: Antigen presentation cells
OMV: Outer membrane vesicles
OMC: Outer membrane compounds
MD: Molecular dynamics

## References

[1] Ainsworth S, Ketter PM, Yu JJ, Grimm RC, May HC, Cap AP, Chambers JP, Guentzel MN, Arulanandam BP (2017). Vaccination with a live attenuated Acinetobacter baumannii deficient in thioredoxin provides protection against systemic Acinetobacter infection. Vaccine.

[2] Berger, S., Lowe, P., Tesar, M., 2015. In: Schmidt, Stefan R., Fusion protein Technologies for Biopharmaceuticals: applications and challenges. MAbs 7. pp. 456–460.

[3] Gharaghie T, Shandiz SAS (2018). The inhibitory effects of silver nanoparticles on bap gene expression in antibiotic-resistant Acientobacter bumanni isolates using real-time PCR. Sci J Ilam Univ Med Sci..

[4] Hajighahramani N, Nezafat N, Eslami M, Negahdaripour M, Rahmatabadi SS, Ghasemi Y (2017). Immunoinformatics analysis and in silico designing of a novel multi-epitope peptide vaccine against Staphylococcus aureus. Infect Genet Evol..

[5] Livingston B, Crimi C, Newman M, Higashimoto Y, Appella E, Sidney J, Sette A (2002). A rational strategy to design multiepitope immunogens based on multiple Th lymphocyte epitopes. J Immunol..

[6] Mahmoodi S, Nezafat N, Barzegar A, Negahdaripour M, Nikanfar AR, Zarghami N, Ghasemi Y (2016). Harnessing bioinformatics for designing a novel multiepitope peptide vaccine against breast cancer. Curr Pharm Biotechnol..

[7] Moise L, Gutierrez A, Kibria F, Martin R, Tassone R, Liu R, Terry F, Martin B, De Groot AS (2015). IVAX: an integrated toolkit for the selection and optimization of antigens and the design of epitope-driven vaccines. Hum Vaccin Immunother..

[8] Negahdaripour M, Eslami M, Nezafat N, Hajighahramani N, Ghoshoon MB, Shoolian E, Dehshahri A, Erfani N, Morowvat MH, Ghasemi Y (2017). A novel HPV prophylactic peptide vaccine, designed by immunoinformatics and structural vaccinology approaches. Infect Genet Evol..

[9] Negahdaripour M, Nezafat N, Eslami M (2018). Structural vaccinology considerations for in silico designing of a multi-epitope vaccine. Infect Genet Evol..

[10] Nezafat N, Eslami M, Negahdaripour M, Rahbar MR, Ghasemi Y (2017). Designing an efficient multi-epitope oral vaccine against helicobacter pylori using immunoinformatics and structural vaccinology approaches. Mol BioSyst..

[11] Ayobami O, Willrich N, Harder T, Okeke IN, Eckmanns T, Markwart R (2019). The incidence and prevalence of hospital-acquired (carbapenem-resistant) Acinetobacter baumannii in Europe, eastern Mediterranean and Africa: a systematic review and meta-analysis. Emerg Microbes Infect..

[12] Piri-Gharaghie T, Doosti A, Mirzaei SA (2023). Novel adjuvant nano-vaccine induced immune response against Acinetobacter baumannii. AMB Express..

[13] Nezafat N, Karimi Z, Eslami M, Mohkam M, Zandian S, Ghasemi Y (2016). Designing an efficient multi-epitope peptide vaccine against vibrio cholerae via combined immunoinformatics and protein interaction-based approaches. Comput Biol Chem..

[14] Piri Gharaghie T, Sadat Shandiz SA, Beiranvand S. Evaluation of silver nanoparticles effects on Bla-per1 gene expression for biofilm formation in isolates of antibiotic-resistant Acientobacter Bumanni by real time PCR method. Cell Mol Res (Iranian Journal of Biology). 2020;35.

[15] Piri-Gharaghie T, Beiranvand S, Riahi A, Shirin NJ, Badmasti F, Mirzaie A, Elahianfar Y, Ghahari S, Ghahari S, Pasban K, Hajrasouliha S (2022). Fabrication and characterization of thymol-loaded chitosan nanogels: improved antibacterial and anti-biofilm activities with negligible cytotoxicity. Chem Biodivers..

[16] Piri-Gharaghie T, Doosti A, Mirzaei SA (2022). Identification of antigenic properties of Acinetobacter baumannii proteins as novel putative vaccine candidates using reverse vaccinology approach. Appl Biochem Biotechnol..

[17] Ma C, Chen W (2021). Where are we and how far is there to go in the development of an Acinetobacter vaccine?. Expert Rev Vaccines.

[18] Piri-Gharaghie T, Doosti A, Mirzaei SA (2022). Fabrication and characterization of pcDNA3. 1 (+) location within chitosan/nanoparticles complexes for enhanced gene delivery. Iran J Biotechnol..

[19] Reygaert WC (2018). An overview of the antimicrobial resistance mechanisms of bacteria. AIMS Microbiol..

[20] Yu K, Liu C, Kim B-G, Lee D-Y (2015). Synthetic fusion protein design and applications. Biotechnol Adv..

[21] Chaudhary N, Weissman D, Whitehead KA (2021). mRNA vaccines for infectious diseases: principles, delivery and clinical translation. Nat Rev Drug Discov..

[22] Singh R, Capalash N, Sharma P (2022). Vaccine development to control the rising scourge of antibiotic-resistant Acinetobacter baumannii: a systematic review. 3 Biotech..

[23] Czerkinsky C, Holmgren J (2010). Topical immunization strategies. Mucosal Immunol..

[24] Saade F, Petrovsky N (2012). Technologies for enhanced efficacy of DNA vaccines. Expert Rev Vaccines.

[25] Vaumourin E, Vourc’h G, Gasqui P, Vayssier-Taussat M (2015). The importance of multiparasitism: examining the consequences of co-infections for human and animal health. Parasit Vectors..

[26] Rehman A, Ahmad S, Shahid F, Albutti A, Alwashmi AS, Aljasir MA, Alhumeed N, Qasim M, Ashfaq UA, Tahir ul Qamar, M. (2021). Integrated core proteomics, subtractive proteomics, and immunoinformatics investigation to unveil a potential multi-epitope vaccine against schistosomiasis. Vaccines.

[27] Sanches RC, Tiwari S, Ferreira LC, Oliveira FM, Lopes MD, Passos MJ, Maia EH, Taranto AG, Kato R, Azevedo VA, Lopes DO (2021). Immunoinformatics design of multi-epitope peptide-based vaccine against Schistosoma mansoni using transmembrane proteins as a target. Front Immunol..

[28] Rawal K, Sinha R, Abbasi BA, Chaudhary A, Nath SK, Kumari P, Preeti P, Saraf D, Singh S, Mishra K, Gupta P (2021). Identification of vaccine targets in pathogens and design of a vaccine using computational approaches. Sci Rep..

[29] Jin JS, Kwon SO, Moon DC, Gurung M, Lee JH, Kim SI, Lee JC (2011). Acinetobacter baumannii secretes cytotoxic outer membrane protein a via outer membrane vesicles. PLoS One..

[30] Cheung RCF, Wong JH, Ng TB (2012). Immobilized metal ion affinity chromatography: a review on its applications. Appl Microbiol Biotechnol..

[31] Dolma KG (2022). Acinetobacter baumannii: an overview of emerging multidrug-resistant pathogen. Med J Malaysia..

[32] Sami SA, Marma KKS, Mahmud S, Khan MAN, Albogami S, El-Shehawi AM, Rakib A, Chakraborty A, Mohiuddin M, Dhama K, Uddin MMN (2021). Designing of a multi-epitope vaccine against the structural proteins of Marburg virus exploiting the immunoinformatics approach. ACS Omega..

