# Supplementary material for: Putative novel outer membrane antigens multi-epitope DNA vaccine candidates identified by Immunoinformatic approaches to control Acinetobacter baumannii
Source: BMC Immunol. 2023 Nov 18;24:46. doi: 10.1186/s12865-023-00585-w (PMC10657578; doi:10.1186/s12865-023-00585-w)
Supplement: Supplementary file 1 — Additional file 1. The information is presented in online version. [file 12865_2023_585_MOESM1_ESM.docx]

**Supplementary materials**

##### **Table S1.** Biological Process Predictions using FFPred Predictions.

| GO term | Name | Prob | SVM Reliability |
| --- | --- | --- | --- |
| GO:0003676 | nucleic acid binding | 0.918 | H |
| GO:0008092 | cytoskeletal protein binding | 0.894 | H |
| GO:0003723 | RNA binding | 0.801 | H |
| GO:0003779 | actin binding | 0.766 | H |
| GO:0019900 | kinase binding | 0.704 | H |
| GO:0015075 | ion transmembrane transporter activity | 0.682 | H |
| GO:0016740 | transferase activity | 0.667 | H |
| GO:0000166 | nucleotide binding | 0.652 | H |
| GO:0003677 | DNA binding | 0.590 | H |
| GO:0003824 | catalytic activity | 0.576 | H |
| GO:0019901 | protein kinase binding | 0.573 | H |
| GO:0044822 | poly(A) RNA binding | 0.553 | H |
| GO:0003700 | sequence-specific DNA binding transcription factor activity | 0.544 | H |
| GO:0046914 | transition metal ion binding | 0.538 | H |
| GO:0046872 | metal ion binding | 0.878 | L |
| GO:0097159 | organic cyclic compound binding | 0.762 | L |
| GO:0043169 | cation binding | 0.731 | L |
| GO:0032403 | protein complex binding | 0.711 | L |
| GO:0005102 | receptor binding | 0.691 | L |
| GO:0019904 | protein domain specific binding | 0.687 | L |
| GO:0036094 | small molecule binding | 0.656 | L |
| GO:0008134 | transcription factor binding | 0.528 | L |
| GO:0070062 | extracellular vesicular exosome | 0.623 | H |
| GO:0071944 | cell periphery | 0.501 | H |
| GO:0043229 | intracellular organelle | 0.933 | L |
| GO:0005737 | cytoplasm | 0.894 | L |
| GO:0043231 | intracellular membrane-bounded organelle | 0.886 | L |
| GO:0005634 | nucleus | 0.790 | L |
| GO:0031981 | nuclear lumen | 0.758 | L |
| GO:0005654 | nucleoplasm | 0.686 | L |
| GO:0005829 | cytosol | 0.674 | L |
| GO:0043234 | protein complex | 0.660 | L |
| GO:0032991 | macromolecular complex | 0.563 | L |

**Table S2.** Global features of vaccine are calculated directly from sequence.

| Feature Name | Value |
| --- | --- |
| aliphatic index | 43.14 |
| atomC | 0.31 |
| atomH | 0.48 |
| atomN | 0.09 |
| atomO | 0.11 |
| atomS | 0.00 |
| charge | -17.02 |
| fraction negative residues | 0.11 |
| fraction positive residues | 0.07 |
| hydrophobicity | -0.62 |
| isoelectric point | 4.33 |
| length | 417.00 |
| molar extinction coefficient | 52770.00 |
| molecular weight | 40996.10 |
| number of atoms | 5487.00 |
| surface area | 72411.00 |
| volume | 47305.90 |

**Table S3.** MolProbity Results of Ramachandran plot.

| MolProbity Score | 1.54 |  |
| --- | --- | --- |
| Clash Score | 0.00 |  |
| Ramachandran Favoured | 100.00% |  |
| Ramachandran Outliers | 0.00% |  |
| Rotamer Outliers | 23.08% | A253 VAL, A270 ILE, A256 GLN, A257 GLU, A242 LEU, A261 ARG |
| C-Beta Deviations | 1 | A239 ASP |
| Bad Bonds | 0 / 259 |  |
| Bad Angles | 3 / 347 | A238 ASN, A265 ASN, A239 ASP |

### **Table S4**. Prediction of discontinuous B-cell epitopes from protein 3D-structure

| A 17 ILE 2 -9.468 -8.609 |
| --- |
| A 18 PRO 19 -11.823 -12.648 |
| A 19 VAL 18 -11.887 -12.590 |
| A 20 GLY 24 -13.098 -14.352 |
| A 21 ALA 11 -15.574 -15.048 |
| A 22 ARG 36 -16.239 -18.512 |
| A 23 ALA 4 -14.681 -13.453 |
| A 24 GLU 35 -17.050 -19.114 |
| A 25 VAL 2 -14.125 -12.730 |
| A 26 GLY 30 -12.971 -14.929 |
| A 27 THR 17 -8.246 -9.253 |
| A 28 THR 38 -8.856 -12.207 |
| A 29 GLY 27 -8.405 -10.543 |
| A 30 TYR 10 -10.191 -10.169 |
| A 31 GLY 22 -10.983 -12.250 |
| A 32 GLY 12 -13.236 -13.094 |
| A 33 ALA 38 -15.039 -17.679 |
| A 34 LEU 6 -16.375 -15.182 |
| A 35 LEU 30 -17.653 -19.073 |
| A 36 TRP 11 -15.204 -14.720 |
| A 37 GLN 21 -12.899 -13.830 |
| A 38 ALA 11 -11.133 -11.117 |
| A 39 ASN 4 -8.910 -8.345 |
| A 40 PRO 11 -9.328 -9.521 |
| A 41 TYR 2 -10.848 -9.831 |
| A 42 VAL 3 -14.300 -13.000 |
| A 43 GLY 33 -16.872 -18.727 |
| A 44 LEU 11 -17.874 -17.083 |
| A 45 ALA 40 -18.347 -20.837 |
| A 46 LEU 7 -15.821 -14.806 |
| A 47 GLY 38 -14.788 -17.458 |
| A 48 TYR 5 -12.327 -11.484 |
| A 49 ASN 30 -9.608 -11.953 |
| A 50 GLY 8 -6.065 -6.287 |
| A 51 GLY 21 -1.029 -3.325 <=B |
| A 52 ASP 0 1.963 1.737 <=B |
| A 53 ILE 20 2.414 -0.164 <=B |
| A 54 SER 7 2.701 1.586 <=B |
| A 55 TRP 8 2.133 0.968 <=B |
| A 56 THR 0 3.784 3.348 <=B |
| A 57 ASP 11 3.603 1.923 <=B |
| A 65 LYS 8 2.360 1.168 <=B |
| A 66 TYR 12 1.751 0.170 <=B |
| A 67 ASP 15 3.621 1.479 <=B |
| A 68 LEU 6 3.820 2.691 <=B |
| A 69 ASP 17 3.347 1.007 <=B |
| A 70 MET 18 2.027 -0.276 <=B |
| A 71 ASP 10 2.832 1.356 <=B |
| A 72 ASN 28 -1.943 -4.940 |
| A 73 ASN 8 -2.828 -3.422 <=B |
| A 74 ASN 37 -7.060 -10.503 |
| A 75 VAL 4 -10.819 -10.035 |
| A 76 TYR 46 -13.779 -17.484 |
| A 77 LEU 5 -15.642 -14.418 |
| A 78 ASN 32 -17.375 -19.057 |
| A 79 ALA 10 -17.840 -16.938 |
| A 80 GLU 38 -18.287 -20.554 |
| A 81 ILE 9 -15.736 -14.962 |
| A 82 ARG 28 -13.768 -15.404 |
| A 83 PRO 11 -10.065 -10.172 |
| A 84 TRP 15 -9.347 -9.997 |
| A 85 GLY 18 -10.429 -11.299 |
| A 86 ALA 3 -5.028 -4.795 |
| A 87 SER 18 -4.460 -6.017 |
| A 88 THR 0 -1.411 -1.248 <=B |
| A 89 ASN 8 -4.292 -4.718 |
| A 90 PRO 25 -8.621 -10.504 |
| A 91 TRP 16 -12.450 -12.859 |
| A 92 ALA 9 -10.817 -10.608 |
| A 93 GLN 19 -11.603 -12.454 |
| A 94 GLY 29 -17.567 -18.882 |
| A 95 LEU 6 -19.493 -17.941 |
| A 96 TYR 33 -19.156 -20.748 |
| A 97 ILE 8 -18.756 -17.519 |
| A 98 ALA 35 -17.514 -19.524 |
| A 99 ALA 6 -15.548 -14.450 |
| A 100 GLY 29 -14.491 -16.160 |
| A 101 ALA 7 -11.911 -11.347 |
| A 102 ALA 38 -10.569 -13.723 |
| A 103 TYR 9 -5.156 -5.598 |
| A 104 LEU 31 -4.101 -7.195 |
| A 105 ASP 11 0.123 -1.156 <=B |
| A 106 ASN 34 1.302 -2.758 <=B |
| A 107 ASP 16 4.348 2.008 <=B |
| A 108 TYR 16 4.741 2.356 <=B |
| A 109 ASP 22 6.064 2.837 <=B |
| A 110 LEU 12 5.185 3.209 <=B |
| A 111 ALA 17 4.119 1.691 <=B |
| A 112 LYS 22 3.342 0.428 <=B |
| A 113 ARG 11 1.300 -0.115 <=B |
| A 114 ILE 25 1.603 -1.456 <=B |
| A 115 GLY 3 1.927 1.361 <=B |
| A 116 ASN 2 2.419 1.911 <=B |
| A 117 GLY 0 2.475 2.191 <=B |
| A 118 ASP 18 1.651 -0.608 <=B |
| A 119 THR 11 1.774 0.305 <=B |
| A 120 LEU 29 1.898 -1.655 <=B |
| A 121 SER 6 2.289 1.335 <=B |
| A 122 ILE 39 3.686 -1.222 <=B |
| A 123 ASP 23 3.792 0.711 <=B |
| A 124 GLY 5 3.126 2.192 <=B |
| A 125 LYS 22 2.437 -0.374 <=B |
| A 126 ASN 8 1.548 0.450 <=B |
| A 127 TYR 31 0.779 -2.875 <=B |
| A 128 GLN 11 1.345 -0.075 <=B |
| A 129 GLN 11 1.439 0.008 <=B |
| A 130 ALA 12 0.505 -0.933 <=B |
| A 131 VAL 2 1.104 0.747 <=B |
| A 132 PRO 2 2.407 1.900 <=B |
| A 133 GLY 1 2.489 2.088 <=B |
| A 134 GLN 10 1.180 -0.105 <=B |
| A 135 GLU 10 1.973 0.596 <=B |
| A 136 GLY 25 1.070 -1.928 <=B |
| A 137 GLY 18 1.402 -0.829 <=B |
| A 138 VAL 35 2.429 -1.875 <=B |
| A 139 ARG 9 4.540 2.983 <=B |
| A 140 GLY 25 5.492 1.985 <=B |
| A 141 LYS 11 5.889 3.947 <=B |
| A 142 MET 25 5.871 2.321 <=B |
| A 143 SER 9 6.056 4.325 <=B |
| A 144 TYR 34 3.174 -1.101 <=B |
| A 145 LYS 7 3.152 1.984 <=B |
| A 146 ASN 24 0.956 -1.914 <=B |
| A 147 ASP 0 -0.807 -0.714 <=B |
| A 148 ILE 6 -4.256 -4.456 |
| A 149 ALA 31 -4.704 -7.728 |
| A 150 PRO 5 -8.669 -8.247 |
| A 151 TYR 35 -10.525 -13.339 |
| A 152 LEU 4 -12.230 -11.284 |
| A 153 GLY 35 -15.876 -18.075 |
| A 154 PHE 3 -20.030 -18.071 |
| A 155 GLY 40 -22.905 -24.871 |
| A 156 PHE 14 -23.432 -22.348 |
| A 157 ALA 40 -22.122 -24.178 |
| A 158 PRO 19 -17.178 -17.387 |
| A 159 LYS 20 -11.417 -12.404 |
| A 160 ILE 23 -8.338 -10.024 |
| A 161 SER 10 -4.033 -4.720 |
| A 162 LYS 2 -4.884 -4.553 |
| A 163 ASN 1 -5.736 -5.192 |
| A 164 TRP 9 -9.043 -9.038 |
| A 165 GLY 28 -14.898 -16.404 |
| A 166 VAL 19 -19.637 -19.564 |
| A 167 PHE 41 -22.615 -24.730 |
| A 168 GLY 13 -23.263 -22.083 |
| A 169 GLU 41 -21.014 -23.313 |
| A 170 VAL 3 -18.182 -16.436 |
| A 171 GLY 28 -12.487 -14.271 |
| A 172 ALA 12 -9.856 -10.103 |
| A 173 TYR 29 -6.923 -9.462 |
| A 174 TYR 8 -1.814 -2.526 <=B |
| A 175 THR 32 0.692 -3.068 <=B |
| A 176 GLY 13 4.534 2.517 <=B |
| A 177 ASN 13 5.689 3.540 <=B |
| A 178 PRO 25 5.890 2.338 <=B |
| A 179 LYS 12 6.438 4.317 <=B |
| A 180 VAL 23 5.966 2.635 <=B |
| A 181 GLU 15 5.844 3.447 <=B |
| A 182 LEU 20 4.225 1.439 <=B |
| A 183 THR 6 2.988 1.954 <=B |
| A 184 GLN 29 2.062 -1.510 <=B |
| A 185 TYR 19 1.069 -1.239 <=B |
| A 186 ASN 16 0.707 -1.214 <=B |
| A 187 LEU 29 0.189 -3.167 <=B |
| A 188 ALA 11 0.477 -0.843 <=B |
| A 189 PRO 11 1.059 -0.328 <=B |
| A 190 VAL 15 0.751 -1.060 <=B |
| A 191 THR 1 1.728 1.414 <=B |
| A 192 GLY 0 1.415 1.252 <=B |
| A 193 ASN 23 1.632 -1.201 <=B |
| A 194 PRO 1 1.033 0.800 <=B |
| A 195 THR 17 0.827 -1.223 <=B |
| A 196 SER 8 0.701 -0.300 <=B |
| A 197 ALA 24 0.894 -1.969 <=B |
| A 198 GLN 0 1.487 1.316 <=B |
| A 199 ASP 8 2.082 0.923 <=B |
| A 200 ALA 25 2.620 -0.557 <=B |
| A 201 VAL 23 3.420 0.382 <=B |
| A 202 ASP 5 3.025 2.102 <=B |
| A 203 LYS 11 3.870 2.160 <=B |
| A 204 GLU 23 4.906 1.697 <=B |
| A 205 ALA 17 5.400 2.824 <=B |
| A 206 ASN 6 6.166 4.767 <=B |
| A 207 GLU 11 5.482 3.586 <=B |
| A 208 ILE 20 5.894 2.916 <=B |
| A 209 ARG 12 6.520 4.391 <=B |
| A 210 ASN 6 6.659 5.203 <=B |
| A 211 ASP 11 6.140 4.169 <=B |
| A 212 ASN 0 4.201 3.718 <=B |
| A 213 LYS 3 3.160 2.452 <=B |
| A 214 TYR 16 4.015 1.713 <=B |
| A 215 GLU 7 4.140 2.859 <=B |
| A 216 TRP 11 -0.038 -1.299 <=B |
| A 217 MET 32 -2.802 -6.160 |
| A 218 PRO 4 -6.067 -5.829 |
| A 219 VAL 42 -10.927 -14.500 |
| A 220 GLY 3 -15.470 -14.036 |
| A 221 LYS 33 -17.404 -19.198 |
| A 222 VAL 6 -20.515 -18.846 |
| A 223 GLY 29 -18.851 -20.018 |
| A 224 VAL 9 -18.801 -17.674 |
| A 225 ASN 30 -17.185 -18.659 |
| A 226 PHE 9 -11.179 -10.928 |
| A 227 TYR 17 -10.084 -10.879 |
| A 228 TRP 9 -6.995 -7.226 |
| B 17 ILE 7 -10.482 -10.081 |
| B 18 PRO 11 -12.600 -12.416 |
| B 19 VAL 18 -12.026 -12.713 |
| B 20 GLY 22 -12.576 -13.660 |
| B 21 ALA 9 -14.769 -14.105 |
| B 22 ARG 37 -14.329 -16.936 |
| B 23 ALA 5 -12.732 -11.843 |
| B 24 GLU 36 -13.133 -15.763 |
| B 25 VAL 2 -10.727 -9.724 |
| B 26 GLY 30 -9.751 -12.080 |
| B 27 THR 18 -7.124 -8.375 |
| B 28 THR 37 -7.701 -11.071 |
| B 29 GLY 27 -7.148 -9.431 |
| B 30 TYR 11 -9.201 -9.408 |
| B 31 GLY 22 -10.656 -11.961 |
| B 32 GLY 11 -13.176 -12.926 |
| B 33 ALA 37 -15.171 -17.681 |
| B 34 LEU 5 -17.038 -15.654 |
| B 35 LEU 31 -19.446 -20.774 |
| B 36 TRP 10 -17.499 -16.637 |
| B 37 GLN 18 -14.655 -15.039 |
| B 38 ALA 14 -15.534 -15.357 |
| B 39 ASN 3 -10.620 -9.743 |
| B 40 PRO 5 -10.240 -9.637 |
| B 41 TYR 2 -13.652 -12.312 |
| B 42 VAL 4 -18.538 -16.866 |
| B 43 GLY 31 -20.726 -21.908 |
| B 44 LEU 10 -21.676 -20.333 |
| B 45 ALA 41 -20.797 -23.121 |
| B 46 LEU 9 -17.968 -16.937 |
| B 47 GLY 37 -15.977 -18.394 |
| B 48 TYR 5 -12.240 -11.407 |
| B 49 ASN 30 -9.140 -11.539 |
| B 50 GLY 9 -5.494 -5.897 |
| B 51 GLY 22 -1.504 -3.861 |
| B 52 ASP 0 2.161 1.912 <=B |
| B 53 ILE 19 1.846 -0.551 <=B |
| B 54 SER 8 2.115 0.952 <=B |
| B 55 TRP 8 1.161 0.107 <=B |
| B 56 THR 4 1.815 1.147 <=B |
| B 57 ASP 11 2.671 1.099 <=B |
| B 58 ASP 3 1.195 0.713 <=B |
| B 59 VAL 17 0.849 -1.204 <=B |
| B 60 SER 12 0.993 -0.501 <=B |
| B 61 VAL 0 1.446 1.280 <=B |
| B 62 ASN 16 1.155 -0.818 <=B |
| B 63 GLY 12 1.054 -0.447 <=B |
| B 64 THR 16 0.617 -1.294 <=B |
| B 65 LYS 19 0.118 -2.080 <=B |
| B 66 TYR 9 0.632 -0.476 <=B |
| B 67 ASP 17 2.435 0.200 <=B |
| B 68 LEU 7 3.194 2.022 <=B |
| B 69 ASP 16 2.679 0.531 <=B |
| B 70 MET 19 1.426 -0.923 <=B |
| B 71 ASP 9 3.247 1.839 <=B |
| B 72 ASN 28 -1.589 -4.626 |
| B 73 ASN 8 -2.230 -2.894 <=B |
| B 74 ASN 38 -7.947 -11.403 |
| B 75 VAL 7 -12.086 -11.501 |
| B 76 TYR 44 -15.487 -18.766 |
| B 77 LEU 13 -18.643 -17.994 |
| B 78 ASN 32 -21.581 -22.779 |
| B 79 ALA 17 -22.406 -21.784 |
| B 80 GLU 35 -21.557 -23.103 |
| B 81 ILE 9 -18.960 -17.815 |
| B 82 ARG 21 -14.554 -15.295 |
| B 83 PRO 9 -11.126 -10.882 |
| B 84 TRP 10 -8.531 -8.700 |
| B 92 ALA 4 -5.820 -5.610 |
| B 93 GLN 11 -8.721 -8.983 |
| B 94 GLY 18 -12.088 -12.768 |
| B 95 LEU 3 -13.099 -11.938 |
| B 96 TYR 30 -16.480 -18.035 |
| B 97 ILE 8 -19.706 -18.360 |
| B 98 ALA 35 -20.621 -22.275 |
| B 99 ALA 12 -19.041 -18.231 |
| B 100 GLY 30 -18.888 -20.166 |
| B 101 ALA 24 -15.263 -16.268 |
| B 102 ALA 38 -11.968 -14.962 |
| B 103 TYR 15 -6.086 -7.111 |
| B 104 LEU 32 -4.554 -7.710 |
| B 105 ASP 10 1.011 -0.255 <=B |
| B 106 ASN 34 1.926 -2.205 <=B |
| B 107 ASP 14 4.705 2.554 <=B |
| B 108 TYR 20 4.591 1.763 <=B |
| B 109 ASP 17 4.996 2.467 <=B |
| B 110 LEU 17 4.526 2.051 <=B |
| B 111 ALA 19 2.840 0.328 <=B |
| B 112 LYS 24 1.861 -1.113 <=B |
| B 113 ARG 19 0.649 -1.611 <=B |
| B 114 ILE 25 0.925 -2.057 <=B |
| B 115 GLY 10 1.284 -0.014 <=B |
| B 116 ASN 5 1.877 1.087 <=B |
| B 117 GLY 4 1.913 1.233 <=B |
| B 118 ASP 24 0.989 -1.884 <=B |
| B 119 THR 11 0.913 -0.457 <=B |
| B 120 LEU 33 0.143 -3.669 <=B |
| B 121 SER 5 1.744 0.968 <=B |
| B 122 ILE 39 3.474 -1.411 <=B |
| B 123 ASP 26 3.309 -0.061 <=B |
| B 124 GLY 5 2.940 2.027 <=B |
| B 125 LYS 23 1.784 -1.066 <=B |
| B 126 ASN 8 0.858 -0.161 <=B |
| B 127 TYR 31 0.420 -3.193 <=B |
| B 128 GLN 12 0.944 -0.545 <=B |
| B 129 GLN 13 1.088 -0.532 <=B |
| B 130 ALA 12 0.315 -1.101 <=B |
| B 131 VAL 1 0.932 0.710 <=B |
| B 132 PRO 3 2.128 1.538 <=B |
| B 133 GLY 1 2.155 1.792 <=B |
| B 134 GLN 8 0.803 -0.210 <=B |
| B 135 GLU 18 1.584 -0.668 <=B |
| B 136 GLY 26 0.649 -2.415 <=B |
| B 137 GLY 23 0.927 -1.825 <=B |
| B 138 VAL 35 2.108 -2.159 <=B |
| B 139 ARG 10 3.851 2.258 <=B |
| B 140 GLY 27 5.231 1.524 <=B |
| B 141 LYS 11 6.014 4.058 <=B |
| B 142 MET 27 5.397 1.671 <=B |
| B 143 SER 7 6.810 5.222 <=B |
| B 144 TYR 35 4.569 0.018 <=B |
| B 145 LYS 8 3.503 2.180 <=B |
| B 146 ASN 28 0.721 -2.582 <=B |
| B 147 ASP 15 -0.802 -2.435 <=B |
| B 148 ILE 26 -6.136 -8.420 |
| B 149 ALA 31 -6.641 -9.442 |
| B 150 PRO 20 -11.296 -12.297 |
| B 151 TYR 35 -13.194 -15.702 |
| B 152 LEU 5 -14.599 -13.495 |
| B 153 GLY 35 -15.347 -17.607 |
| B 154 PHE 2 -17.381 -15.612 |
| B 155 GLY 39 -16.614 -19.188 |
| B 156 PHE 2 -14.469 -13.035 |
| B 157 ALA 38 -14.727 -17.403 |
| B 158 PRO 2 -11.143 -10.092 |
| B 159 LYS 14 -8.961 -9.541 |
| B 160 ILE 11 -6.179 -6.733 |
| B 161 SER 4 -4.821 -4.727 |
| B 162 LYS 0 -5.700 -5.045 |
| B 163 ASN 0 -6.469 -5.725 |
| B 164 TRP 2 -8.310 -7.585 |
| B 165 GLY 23 -10.559 -11.990 |
| B 166 VAL 6 -12.578 -11.821 |
| B 167 PHE 39 -15.000 -17.760 |
| B 168 GLY 2 -14.371 -12.949 |
| B 169 GLU 41 -14.599 -17.635 |
| B 170 VAL 2 -13.146 -11.864 |
| B 171 GLY 29 -11.196 -13.243 |
| B 172 ALA 12 -8.781 -9.151 |
| B 173 TYR 29 -8.261 -10.646 |
| B 174 TYR 15 -3.037 -4.413 |
| B 175 THR 31 0.366 -3.241 <=B |
| B 176 GLY 15 5.407 3.060 <=B |
| B 177 ASN 13 7.204 4.880 <=B |
| B 178 PRO 25 7.155 3.457 <=B |
| B 179 LYS 11 7.214 5.119 <=B |
| B 180 VAL 23 6.038 2.699 <=B |
| B 181 GLU 13 5.526 3.395 <=B |
| B 182 LEU 22 4.315 1.289 <=B |
| B 183 THR 7 3.040 1.885 <=B |
| B 184 GLN 30 1.828 -1.833 <=B |
| B 185 TYR 12 0.714 -0.748 <=B |
| B 186 ASN 17 0.433 -1.572 <=B |
| B 187 LEU 30 -0.062 -3.504 <=B |
| B 188 ALA 11 0.764 -0.589 <=B |
| B 189 PRO 11 0.859 -0.504 <=B |
| B 190 VAL 14 0.679 -1.009 <=B |
| B 191 THR 0 1.442 1.276 <=B |
| B 192 GLY 0 1.409 1.247 <=B |
| B 193 ASN 24 1.175 -1.720 <=B |
| B 194 PRO 1 1.031 0.798 <=B |
| B 195 THR 17 0.899 -1.159 <=B |
| B 196 SER 8 0.656 -0.339 <=B |
| B 197 ALA 24 1.147 -1.745 <=B |
| B 198 GLN 0 1.408 1.246 <=B |
| B 199 ASP 8 2.166 0.997 <=B |
| B 200 ALA 25 2.713 -0.474 <=B |
| B 201 VAL 24 3.430 0.275 <=B |
| B 202 ASP 5 2.829 1.929 <=B |
| B 203 LYS 12 3.949 2.115 <=B |
| B 204 GLU 23 4.859 1.655 <=B |
| B 205 ALA 17 5.432 2.852 <=B |
| B 206 ASN 5 6.524 5.199 <=B |
| B 207 GLU 12 5.536 3.519 <=B |
| B 208 ILE 22 5.940 2.727 <=B |
| B 209 ARG 12 7.086 4.891 <=B |
| B 210 ASN 6 7.421 5.878 <=B |
| B 211 ASP 12 6.558 4.424 <=B |
| B 212 ASN 0 4.511 3.992 <=B |
| B 213 LYS 6 3.967 2.821 <=B |
| B 214 TYR 16 4.813 2.419 <=B |
| B 215 GLU 9 4.222 2.701 <=B |
| B 216 TRP 10 0.349 -0.841 <=B |
| B 217 MET 33 -1.666 -5.269 |
| B 218 PRO 4 -5.200 -5.062 |
| B 219 VAL 44 -9.561 -13.521 |
| B 220 GLY 1 -11.475 -10.270 |
| B 221 LYS 33 -12.285 -14.667 |
| B 222 VAL 1 -13.555 -12.111 |
| B 223 GLY 29 -13.265 -15.075 |
| B 224 VAL 6 -13.054 -12.243 |
| B 225 ASN 27 -13.697 -15.227 |
| B 226 PHE 8 -10.634 -10.331 |
| B 227 TYR 17 -9.815 -10.641 |
|  |
| Identified 208 B-Cell epitope residues out of 409 total residues |
